# Estimating three- and four-parameter MIRT models with importance-weighted sampling enhanced variational auto-encoder

**DOI:** 10.3389/fpsyg.2022.935419

**Published:** 2022-08-15

**Authors:** Tianci Liu, Chun Wang, Gongjun Xu

**Affiliations:** ^1^Department of Statistics, University of Michigan, Ann Arbor, MI, United States; ^2^College of Education, University of Washington, Seattle, WA, United States

**Keywords:** Multidimensional Item Response Theory (MIRT), estimation, Monte Carlo (MC) algorithm, variational auto encoder (VAE), four parameter item response theory

## Abstract

Multidimensional Item Response Theory (MIRT) is widely used in educational and psychological assessment and evaluation. With the increasing size of modern assessment data, many existing estimation methods become computationally demanding and hence they are not scalable to big data, especially for the multidimensional three-parameter and four-parameter logistic models (i.e., M3PL and M4PL). To address this issue, we propose an importance-weighted sampling enhanced Variational Autoencoder (VAE) approach for the estimation of M3PL and M4PL. The key idea is to adopt a variational inference procedure in machine learning literature to approximate the intractable marginal likelihood, and further use importance-weighted samples to boost the trained VAE with a better log-likelihood approximation. Simulation studies are conducted to demonstrate the computational efficiency and scalability of the new algorithm in comparison to the popular alternative algorithms, i.e., Monte Carlo EM and Metropolis-Hastings Robbins-Monro methods. The good performance of the proposed method is also illustrated by a NAEP multistage testing data set.

## 1. Introduction

*Item response theory* (IRT) has been widely used for the evaluation and assessment of education and psychology test data. The most commonly used IRT is the *2-parameter logistic model* (2PL), which is based on a logistic model for dichotomous responses and assigns a scalar factor score for each respondent. After observing its success, flexibility beyond the 2PL model has also been pursued for decades. Notably, McDonald (1967) suggested that the lower and upper asymptote in 2PL can be freed up from fixed 0 and 1, respectively. Estimating a different lower asymptote for each item results in the so-called 3PL model, which has been quite useful for multiple-choice items where guessing is possible; but little empirical evidence was found to support that estimating upper asymptote was beneficial as well; therefore, it was widely believed that the 4PL model was only of theoretical interest and there was no compelling reason for practitioners to use it (Barton and Lord, 1981; Hambleton and Swaminathan, 1985). Until the 2000s, researchers started revisiting the 4PL model and demonstrated the rationale of introducing upper asymptote parameters after observing early signs of its importance (Reise and Waller, 2003; Loken and Rulison, 2010; Waller and Reise, 2010; Yen et al., 2012). Waller and Feuerstahler (2017) took a step further and conducted a comprehensive study of 4PL model on a variety of real and synthetic data. In their experiment, the 4PL model achieved promising accuracy on medium to large data. However, despite these existing studies and estimation methods (e.g., Ogasawara, 2002; Waller and Feuerstahler, 2017; Meng et al., 2020), difficulties of parameter estimation in 3PL and 4PL models still remain, especially when data sizes are large and the latent factors exhibit a multidimensional or even high-dimensional structure.

*Multidimensional IRT* (MIRT) models are a family of models where the latent trait is no longer assumed to be unidimensional. By allowing latent factors to exhibit multidimensional structures, 2PL, 3PL, and 4PL models are turned into the *multidimensional 2PL* (M2PL), 3PL (M3PL), and 4PL (M4PL) models, respectively. Compared with IRT models, MIRT models are capable to model each individual's multiple latent traits simultaneously and are usually favored by large scale and complex real data, thereof (Reckase, 2009).

In this article, we study the general MIRT models with a special focus on M3PL and M4PL models. Specifically, assume that there are *N* individuals who respond to *J* items independently with binary response *Y*_*ij*_, for *i* = 1, …, *N* and *j* = 1, …, *J*. The M3PL model assumes that this response from the *i*-th individual to the *j*-th item is modeled by the following *item response function* (IRF).

(1) $$\begin{array}{l} P\left(Y_{ij}=1|\theta_{i};a_{j},b_{j},c_{j}\right)=c_{j}+\left(1-c_{j}\right)\frac{\exp\left(a_{j}^{⊤}\theta_{i}+b_{j}\right)}{\exp\left(a_{j}^{⊤}\theta_{i}+b_{j}\right)+1}, \end{array}$$

where ***a***_*j*_ is a *K*-dimensional vector of item discrimination (loading) parameters for the *j*-th item; *b*_*j*_ is referred to as the item easiness parameters. −*b*_*j*_/||***a***_*j*_||_2_ is sometimes termed as item difficulty (Cho et al., 2021); *c*_*j*_ ≥ 0 is known as the lower asymptote of the *j*-th item and measures the probability of guessing *j*-th item correctly when ***θ***_*i*_ is of negative infinity. Moreover, ***θ***_*i*_ is a *K*-dimensional latent variable denoting the ability of *i*-th respondent, which is assumed to have a standard *K*-dimensional Gaussian distribution in IRT literature. Further generalizing M3PL, the M4PL model has an IRF of

(2) $$\begin{array}{l} P\left(Y_{ij}=1|\theta_{i};a_{j},b_{j},c_{j},d_{j}\right)=c_{j}+\left(d_{j}-c_{j}\right)\frac{\exp\left(a_{j}^{⊤}\theta_{i}+b_{j}\right)}{\exp\left(a_{j}^{⊤}\theta_{i}+b_{j}\right)+1}, \end{array}$$

where additional *d*_*j*_ ≤ 1 is referred to as the upper asymptote parameter, which is the maximum probability of answering the *j*-th item correctly when ***θ***_*i*_ goes to infinity. Intuitively, 1 − *d*_*j*_ can be treated as the slipping probability that an individual who is able to answer the item correctly but miss it accidentally.

For both M3PL and M4PL models, we denote model parameters **A** = {***a***_*j*_, *j* = 1, …, *J*}, ***b*** = {*b*_*j*_, *j* = 1, …, *J*}, ***c*** = {*c*_*j*_, *j* = 1, …, *J*}, ***d*** = {*d*_*j*_, *j* = 1, …, *J*}; and for M3PL model *d*_*j*_ = 1, *j* = 1, …, *J* and *M*_*p*_ = {**A**, ***b***, ***c***, ***d***} is the collection of all model parameters. Under the typical local independence assumption, the marginal log-likelihood of *M*_*p*_ is given by

(3) $$\begin{array}{l} l\left(M_{p};\text{Y}\right)=\overset{N}{\underset{i=1}{\sum }}\log P\left(y_{i}|M_{p}\right)\\ \;=\overset{N}{\underset{i=1}{\sum }}\log\int \overset{J}{\underset{j=1}{\prod }}P\left(Y_{ij}|\theta_{i};M_{p}\right)p\left(\theta_{i}\right)\text{d}\theta_{i}, \end{array}$$

where *p*(***θ***_*i*_) is the probability density function of a standard *K*-dimensional Gaussian distribution.

Due to the latent variable structure, the *K* dimensional integrals involved in (3) makes maximization of the log-likelihood function with respect to *M*_*p*_ intractable. Direct numerical approximations of the integrals were proposed, including the *Gauss-Hermite quadrature* (Bock and Aitkin, 1981) and *Laplace approximation* (Tierney and Kadane, 1986; Lindstrom and Bates, 1988; Wolfinger and O'connell, 1993). However, these methods usually fail to handle complicated MIRT model, especially when the dimension *K* of latent factors ***θ*** grows: Gauss-Hermite quadrature quickly becomes computationally expensive in a high-dimensional setting; the Laplace approximation, though being efficient in computation, often performs less accurately when *K* increases or when the likelihood function is skewed. *Monte Carlo* (MC) simulations have also been applied to obtain numerical approximations for MIRT, such as *Monte Carlo expectation-maximization* (MCEM, McCulloch, 1997), *stochastic expectation-maximization* (StEM, von Davier and Sinharay, 2010), and *Metropolis-Hastings Robbins-Monro* (MHRM, Cai, 2010a,b). Nevertheless, MC based methods need drawing samples from posterior distributions, which could be computationally demanding as well. Recently, Zhang et al. (2020) improved StEM for item factor analysis, but its stochastic E-step involves an adaptive rejection-based Gibbs sampler and may still be time consuming. All methods discussed above can be seen as variants of the *marginal maximum likelihood* (MML) estimator proposed in Bock and Aitkin (1981), where latent ***θ*** are considered as random variables and are integrated out. Chen et al. (2019) instead studied the *constraint joint maximum likelihood estimator* (CJMLE) by treating ***θ*** as fixed effect parameters in order to achieve higher speeds.

Unfortunately, many existing studies focusing on the M2PL model cannot be applied to M3(4)PL models easily: for MHRM, commercial software FlexMIRT (Chung and Houts, 2020) does not support M4PL, and for M3PL, MHRM is known to suffer from a lower convergence rate (Cho et al., 2021) than M2PL; for CJMLE, the authors only derived methods for M2PL and which not support M3(4)PL models. In general, computationally efficient estimation methods for M3(4)PL models are still under explored.

Variational approaches stem from the machine learning literature, which maximizes a tractable lower bound of the log-likelihood rather than maximizing the log-likelihood directly. They have been applied to fitting IRT models in recent years (Rijmen and Jeon, 2013; Natesan et al., 2016; Hui et al., 2017; Jeon et al., 2017). More recently, these variational methods also established a variety of successes on more complicated MIRT (Curi et al., 2019; Wu et al., 2020; Cho et al., 2021) and graded response models (Urban and Bauer, 2021). Notably, *variational autoencoder* (VAE), deep learning based variational method, and its variation, *importance weighted autoencoder* (IWAE), are shown to be effective in parameters estimation and achieve performances competitive to traditional techniques at much faster speeds (Curi et al., 2019; Wu et al., 2020; Urban and Bauer, 2021).

In this article, we investigate the VAE method for the more challenging M3(4)PL models with possible missing data. Extending explorations from Urban and Bauer (2021), we propose a new training strategy for VAE by enhancing it with the objective function of IWAE. As revealed in Section 2.2, although IWAE is computationally more expensive than VAE, our mixing training method inherits both the speed advantage of VAE and the better performance of IWAE. We also pay great attention to several practical issues and challenges in model training and propose corresponding methods/tricks to solve them, which allows our model to handle missing data and have better numeric robustness. Compared with the existing estimation approaches, such as MCEM and MHRM, our method succeeds in achieving comparable or better accuracy in parameter estimation and exhibits a much faster speed. Moreover, our method converges under M3(4)PL models within constant fitting times on different sizes, comparable to what Urban and Bauer (2021) found in the M2PL model, which is a key advantage of VAE based estimation over traditional methods.

The rest of this article is organized as follows. Section 2 covers our new training strategy of VAE based estimation, which is named as *Importance-Weighted sampling enhanced VAE* (IWVAE); to make the section self-contained, we also provide an overview of VAE and IWAE; important tricks for handling missing data as well as improving numerical stability are also introduced. Section 3 provides a large-scale simulation study where IWVAE shows consistently competitive performances to MHRM and MCEM methods across different sample sizes, item structures, and asymptotic regimes. Section 4 compares three methods on a real data set from a multistage testing design. We end up this article with final discussion and remarks in Section 5.

## 2. Methods

We start with a brief overview of variational inference and how it helps tackle maximizing likelihoods whose exact forms are unavailable. We then introduce a gradient based model from deep learning called *variational autoencoder* (VAE), along with its generalization *importance weighted autoencoder* (IWAE). Given the importance and popularity of *multilayer perceptron* (MLP) in machine learning, which provides an efficient way of parameterizing and implementing VAE and IWAE, we include a concise introduction of MLP and reveal its ability to handle missing entries which are ubiquitous in large datasets. We end up this section with our new proposed mixing training method of VAE, *importance-weighted sampling enhanced VAE* (IWVAE). IWVAE uses both VAE and IWAE's objective functions and enjoys both benefits of them.

### 2.1. A review of variational inference and variational autoencoder

Since the integration in Equation (3) does not admit a closed form solution, we need a tractable objective function to approximate it, and *variational inference* (VI) is a machine learning technique to achieve this (Bishop, 2006; Blei et al., 2017). There are two equivalent ways to setup the VI objective. The first one aims to find the best approximation of the posterior of latent variable ***θ***_*i*_ given ***y***_*i*_ and *M*_*p*_, which results in a lower bound of Equation (3). Additionally, the second one directly derives the bound using Jensen Inequality.

We start with the first derivation as it better clarifies the connection between VI and the *expectation maximization* (EM) algorithm (Dempster et al., 1977). The second derivation is revisited in Section 2.2 when we introduce a new tighter lower bound. Let **Θ** = (***θ***_1_, …, ***θ***_*N*_) denote the collection of all latent variables. The *best* approximation of posterior *p*(**Θ** ∣ **Y**; *M*_*p*_), which we refer to as *q*(**Θ**), is obtained by finding a candidate from some simple and tractable variational distribution families such that the *Kullback-Leibler* (KL) divergence *D*_KL_[*q*(**Θ**)||*p*(**Θ**∣**Y**; *M*_*p*_)] is minimized. One common variational family is the factorized distribution $q\left(\Theta \right)=\prod_{i=1}^{N}\prod_{k=1}^{K}q_{ik}\left(\theta_{ik}\right)$, where the subscript *ik* in *q*_*ik*_(θ_*ik*_) is to emphasize that different dimensions can follow different distributions, or follow the same distribution but have different parameters. For instance, we can choose the popular Gaussian distribution for each *q*_*ik*_(θ_*ik*_), equivalently, we have *q*(***θ***_*i*_) to follow a *K*-dimensional diagonal Gaussian distribution. If one intends to characterize the dependence structure among different dimensions of ***θ***_*i*_, we may choose the factorized distribution family $q\left(\Theta \right)=\prod_{i=1}^{N}q_{i}\left(\theta_{i}\right)$, with *q*_*i*_(***θ***_*i*_) following a *K*-dimensional Gaussian distribution.

Under this setting, the optimal variational approximation q^*^(**Θ**) is given by (Blei et al., 2017).

(4) $$\begin{array}{l} q^{*}\left(\Theta \right)≜\underset{q\left(\Theta \right)}{\mathrm{argmin}}D_{\mathrm{KL}}\left[q\left(\Theta \right)||p\left(\Theta |\text{Y};M_{p}\right)\right]\\ \;=\underset{q\left(\Theta \right)}{\mathrm{argmin}}\int q\left(\Theta \right)\log q\left(\Theta \right)\text{d}\Theta \\ \;-\int q\left(\Theta \right)\log\frac{p\left(\text{Y}|\Theta ;M_{p}\right)p\left(\Theta \right)}{p\left(\text{Y}|M_{p}\right)}\text{d}\Theta . \end{array}$$

Note that log *p*(**Y** ∣ *M*_*p*_) is independent of **Θ**, it is easy to obtain the optimization objective

(5) $$\begin{array}{l} q^{*}\left(\Theta \right)=\underset{q\left(\Theta \right)}{\mathrm{argmin}}D_{\mathrm{KL}}\left[q\left(\Theta \right)||p\left(\Theta \right)\right]-{𝔼}_{q\left(\Theta \right)}\left[\log p\left(\text{Y}|\Theta ;M_{p}\right)\right], \end{array}$$

and following decomposition

(6) $$\begin{array}{l} \log p\left(\text{Y}|M_{p}\right)={𝔼}_{q\left(\Theta \right)}\left[\log p\left(\text{Y}|\Theta ;M_{p}\right)\right]-D_{\mathrm{KL}}\left[q\left(\Theta \right)||p\left(\Theta \right)\right]\\ \;+D_{\mathrm{KL}}\left[q\left(\Theta \right)||p\left(\Theta |\text{Y};M_{p}\right)\right]. \end{array}$$

Since *D*_KL_[*q*(**Θ**)||*p*(**Θ** ∣ **Y**; *M*_*p*_)] is non-negative, the decomposition reveals the fact that minimizing Equation (5) is equivalent to maximizing a lower bound of the marginal log-likelihood, which is known as *evidence lower bound* (ELBO).

**Remark 1**. *The derivation of VI above has a close connection to the EM algorithm. Using the decomposition from Bishop (2006), we have*

(7) $$\begin{array}{l} \log p\left(\text{Y}|M_{p}\right)=\int q\left(\Theta \right)\log p\left(\text{Y}|M_{p}\right)d\Theta \\ \;=\int q\left(\Theta \right)\log\frac{p\left(\text{Y},\Theta |M_{p}\right)}{q\left(\Theta \right)}d\Theta \\ \;+\int q\left(\Theta \right)\log\frac{q\left(\Theta \right)}{p\left(\Theta |\text{Y},M_{p}\right)}d\Theta \\ \;=L\left(q\left(\Theta \right),M_{p}\right)+D_{\mathrm{KL}}\left[q\left(\Theta \right)||p\left(\Theta |\text{Y},M_{p}\right)\right], \end{array}$$

*where q*(**Θ**) *is an arbitrary distribution that includes the variational distribution families. And the first term* $L\left(q\left(\Theta \right),M_{p}\right)$ *is precisely the ELBO. In the EM algorithm*, $L\left(q\left(\Theta \right),M_{p}\right)$ *is maximized with respect to q*(**Θ**) *and M*_*p*_ *in an iterative way. In the E-step, the maximization is over q*(**Θ**), *which requires a closed-form solution: the true posterior of* **Θ** given **Y** and fixed $M_{p}^{old}$. *By doing so the second KL divergence disappears and* $L\left(q\left(\Theta \right),M_{p}^{old}\right)=\log p\left(\text{Y}|M_{p}^{old}\right)$. *Since the right hand side does not depend on q*(**Θ**), *the ELBO takes equality thereof. In M-step, the M*_*p*_ *is optimized to maximize the* $L\left(q\left(\Theta \right),M_{p}\right)$ *by fixing q*(**Θ**). *By repeating two steps the EM algorithm is guaranteed to converge to a local optimum of* log *p*(**Y** ∣ *M*_*p*_).

*The main difference between VI on IRT and EM algorithm is that because p*(**Θ** ∣ **Y**; *M*_*p*_) *is intractable, we cannot obtain the analytic update of q*(**Θ**) *in each step, as a result, plain EM algorithm does not scale up well to the high-dimensional MIRT model. VI, on the other hand, finds a tractable approximation in its “E-step” and consequently, it always optimizes a strict lower bound. In general, another philosophical difference between VI and EM is that unknown parameters in VI are usually treated as latent variables as well, refer to Bishop (2006) for more clarifications. In our setup, we distinguish model parameters M*_*p*_ *and latent variable* **Θ**, *but this is not necessary, refer to Wu et al. (2020) where M*_*p*_ *was also treated as latent variables as well and modeled together with* **Θ**.

Evidence lower bound derived in Equation (5) is a global lower bound of the marginal log-likelihood of all observations. Given the local independence assumption, we can obtain a tighter lower bound by constructing each individual a corresponding local lower bound. Deriving local lower bounds indicate finding *q*_*i*_(***θ***_*i*_) such that *D*_KL_[*q*_*i*_(***θ***_*i*_)||*p*(***θ***_*i*_ ∣ ***y***_*i*_; *M*_*p*_)] is minimized. This is called *local variational methods*, we recommend Chapter 10.4 of Bishop (2006) for more detailed explanations, and Cho et al. (2021) for its successful implementations on M2PL and M3PL models.

However, despite the success of Cho et al. (2021), in general, the local variational method is computationally expensive on large scale data. One alternate to handle this challenge is the *amortized variational inference* (AVI). To characterize $q_{i}\left(\theta_{i}\right)=N\left(\mu_{i},\sigma_{i}^{2}\right)$, where $\sigma_{i}^{2}$ denotes the diagonal of the covariance matrix, AVI assumes that $\mu_{i},\sigma_{i}^{2}$ depend on ***y***_*i*_ through a function *F*(·) parameterized by ***ϕ***, formally

(8) $$\begin{array}{l} \left(\mu_{i},\log\sigma_{i}^{2}\right)=F_{\phi }\left(y_{i}\right),\;q_{i}\left(\theta_{i}\right)=N\left(\mu_{i},\sigma_{i}^{2}\right). \end{array}$$

Henceforth, we denote *q*_*i*_(***θ***_*i*_) as *q*_***ϕ***_(***θ***_*i*_ ∣ ***y***_*i*_). In practice, *F*_***ϕ***_ can be flexible and expressed by a deep neural network. One of its most famous applications on AVI is the *variational autoencoder* (VAE) proposed by Kingma and Welling (2014). VAE uses two neural networks together to maximize the ELBO bound: *F*_***ϕ***_ is termed as *inference* or *encoder* network (please refer to Section 2.3.1 for the specification of *F*_***ϕ***_); the other *generative* or *decoder* network learns the generative process of ***y***_*i*_ given ***θ***_*i*_, where this process in MIRT is essentially estimating model parameters *M*_*p*_.

In VAE, ***ϕ*** and *M*_*p*_ are learned through stochastic gradient descents. Following Kingma and Welling (2014) and Urban and Bauer (2021), we give a brief review here. Note the ELBO for the *i*-th individual is given by

(9) $$\begin{array}{l} {\text{ELBO}}_{i}={𝔼}_{q_{\phi }\left(\theta_{i}|y_{i}\right)}\left[\log p\left(y_{i}|\theta_{i};M_{p}\right)\right]-D_{\mathrm{KL}}\left[q_{\phi }\left(\theta_{i}|y_{i}\right)||p\left(\theta_{i}\right)\right] \end{array}$$

The gradient ∇_*M*_*p*__ ELBO_*i*_ can be estimated readily with *S* Monte Carlo samples $\theta_{i}^{s}\sim q_{\phi }\left(\theta_{i}|y_{i}\right)$ for *s* = 1, …, *S* as $\nabla_{M_{p}}{\text{ELBO}}_{i}\approx \frac{1}{S}\overset{S}{\underset{s=1}{\sum }}\nabla_{M_{p}}\log p\left(y_{i}|\theta_{i}^{s};M_{p})\right]$. However, gradient ∇_***ϕ***_ELBO_*i*_ cannot be obtained in the same way, as in general ∇_***ϕ***_ and 𝔼_*q*_***ϕ***_(***θ***_*i*_ ∣ ***y***_*i*_)_ cannot be switched. To solve this problem, Kingma and Welling (2014) reparameterized $\theta_{i}\sim N\left(\mu_{i},\sigma_{i}^{2}\right)$ as follows

(10) $$\begin{array}{l} e_{i}\sim N\left(0,\text{I}\right),\;\theta_{i}=e_{i}⊙\sigma_{i}+\mu_{i}, \end{array}$$

where ⊙ means the element-wise multiplications. By transforming the integration over *q*_***ϕ***_(***θ***_*i*_ ∣ ***y***_*i*_) to *p*(***e***_*i*_), we have

$$\begin{array}{l} \nabla_{\phi }{\text{ELBO}}_{i}=\nabla_{\phi }{𝔼}_{q_{\phi }\left(\theta_{i}|y_{i}\right)}\left[\log p\left(y_{i}|\theta_{i};M_{p}\right)\right]\\ \;-\nabla_{\phi }D_{\mathrm{KL}}\left[q_{\phi }\left(\theta_{i}|y_{i}\right)||p\left(\theta_{i}\right)\right]. \end{array}$$

Then, the first term can be estimated with Monte Carlo samples $\frac{1}{S}\overset{S}{\underset{s=1}{\sum }}\nabla_{\phi }\log p\left(y_{i}|e_{i}^{s}⊙\sigma_{i}+\mu_{i};M_{p}\right)$, and the second term can be computed effectively by observing that the KL divergence between *q*_***ϕ***_(***θ***_*i*_ ∣ ***y***_*i*_) and *p*(***θ***_*i*_) has an analytic form (Kingma and Welling, 2014).

(11) $$\begin{array}{l} D_{\mathrm{KL}}\left[q_{\phi }\left(\theta_{i}|y_{i}\right)||p\left(\theta_{i}\right)\right]=\frac{1}{2}\overset{K}{\underset{k=1}{\sum }}\left(\mu_{ik}+\sigma_{ik}^{2}-1-\log\sigma_{ik}^{2}\right). \end{array}$$

The gradient can be computed readily through the chain rule thereof. For more details, please refer to Kingma and Welling (2014) and Urban and Bauer (2021).

### 2.2. Importance weighted variational inference

Since the ELBO is a lower bound of the marginal likelihood that we want to maximize, a tighter ELBO is appealing as the true likelihood can be approximated more accurately. It is known that the tightness of the ELBO is coupled with the expressiveness of the variational family and limited expressivity can negatively affect the learned models, and there have been many works on reducing the gap between ELBO and marginal log-likelihood (Burda et al., 2016; Kingma et al., 2016; Kingma and Welling, 2019). Some studies aimed to extend the capacity of the variational family, and techniques including normalizing flows have been applied (Kingma et al., 2016; Papamakarios et al., 2021).

Burda et al. (2016) introduced a new *importance-weighted ELBO* (IW-ELBO) which alleviated the coupling without changing the variational families. To better illustrate the connection between IW-ELBO bound and ELBO, we start with the second derivation of ELBO *via* Jensen Inequality.

(12) $$\begin{array}{l} \log p\left(y_{i}|M_{p}\right)=\log{𝔼}_{q_{\phi }\left(\theta_{i}|y_{i}\right)}\left[\frac{p\left(y_{i},\theta_{i}|M_{p}\right)}{q_{\phi }\left(\theta_{i}|y_{i}\right)}\right]\\ \geq {𝔼}_{q_{\phi }\left(\theta_{i}|y_{i}\right)}\left[\log\frac{p\left(y_{i},\theta_{i}|M_{p}\right)}{q_{\phi }\left(\theta_{i}|y_{i}\right)}\right]={\text{ELBO}}_{i}. \end{array}$$

The above derivation can be generalized as follows

(13) $$\begin{array}{l} \log p\left(y_{i}|M_{p}\right)=\log{𝔼}_{\theta_{i}^{1},\ldots ,\theta_{i}^{R}\sim q_{\phi }\left(\theta_{i}|y_{i}\right)}\left[\frac{1}{R}\overset{R}{\underset{r=1}{\sum }}\frac{p\left(y_{i},\theta_{i}^{r}|M_{p}\right)}{q_{\phi }\left(\theta_{i}^{r}|y_{i}\right)}\right]\\ \;\geq {𝔼}_{\theta_{i}^{1:R}\sim q_{\phi }\left(\theta_{i}|y_{i}\right)}\left[\log\frac{1}{R}\overset{R}{\underset{r=1}{\sum }}w_{i}^{r}\right]. \end{array}$$

Equation (13) is known as IW-ELBO where $w_{i}^{r}≜p\left(y_{i},\theta_{i}^{r}|M_{p}\right)/q_{\phi }\left(\theta_{i}^{r}|y_{i}\right)$. When *q*_***ϕ***_ is reparameterizable, Monte Carlo estimates of IW-ELBO and its gradient are given by

(14) $$\begin{array}{l} {𝔼}_{\theta_{i}^{1:R}\sim q_{\phi }\left(\theta_{i}|y_{i}\right)}\left[\log\frac{1}{R}\overset{R}{\underset{r=1}{\sum }}w_{i}^{r}\right]\\ \approx \frac{1}{S}\overset{S}{\underset{s=1}{\sum }}\log\frac{1}{R}\overset{R}{\underset{r=1}{\sum }}w_{i}^{rs}, \end{array}$$

(15) $$\begin{array}{l} \;\nabla_{\phi ,M_{p}}{𝔼}_{\theta_{i}^{1:R}\sim q_{\phi }\left(\theta_{i}|y_{i}\right)}\left[\log\frac{1}{R}\overset{R}{\underset{r=1}{\sum }}w_{i}^{r}\right]\\ ={𝔼}_{e_{i}^{1:R}}\left[\nabla_{\phi ,M_{p}}\log\frac{1}{R}\overset{R}{\underset{r=1}{\sum }}w_{i}^{r}\right]\\ ={𝔼}_{e_{i}^{1:R}}\left[\frac{w_{i}^{r}}{\overset{R}{\underset{r=1}{\sum }}w_{i}^{r}}\nabla_{\phi ,M_{p}}\log w_{i}^{r}\right]\\ \;\approx \frac{1}{S}\overset{S}{\underset{s=1}{\sum }}\overset{R}{\underset{r=1}{\sum }}\frac{w_{i}^{rs}}{\overset{R}{\underset{r=1}{\sum }}w_{i}^{rs}}\nabla_{\phi ,M_{p}}\log w_{i}^{rs}, \end{array}$$

where *S* and *R* are corresponding numbers of Monte Carlo samples and importance-weighted samples. Replacing ELBO with IW-ELBO in VAE leads to IWAE, which is a generalization of the VAE, as indicated by observing that IW-ELBO will reduce to ELBO for *R* = 1. Notably, IW-ELBO increases in *R* and converges to log *p*(***y***_*i*_ ∣ *M*_*p*_) as *R* → ∞ under mild conditions (Burda et al., 2016).

However, Rainforth et al. (2018) showed that using more important samples is not always helpful. The authors introduced the *signal-to-noise ratio* (SNR) of an estimator δ as the ratio between the absolute value of its expectation and its SD, i.e., SNR(δ) ≜ |𝔼(δ)|/σ(δ). Then they show the below orders (rewritten with our notations)

$$\begin{array}{l} \text{SNR}\left(M_{p}\right)=O\left(\sqrt{RS}\right),\;\text{SNR}\left(\phi \right)=O\left(\sqrt{S/R}\right). \end{array}$$

In words, for any given *S*, increasing *R* makes gradient estimates of parameters ***ϕ*** in the inference network noisier. Despite the fact that the estimates of *M*_*p*_ along may benefit from a tighter likelihood bound, the final result can be deteriorated due to the worse inference network as shown in Rainforth et al. (2018).

To mitigate this problem, one simple solution is to increase *S* of the same order, but such modification takes more computational costs and slows down the training. We apply the *doubly reparameterized gradient estimator* (DReG) from Tucker et al. (2018), a recently developed method that gets rid of a similar issue. Specifically, we use the below estimator to update the inference network

(16) $$\begin{array}{l} \;\nabla_{\phi }{𝔼}_{\theta_{i}^{1:R}\sim q_{\phi }\left(\theta_{i}|y_{i}\right)}\left[\log\frac{1}{R}\sum_{r=1}^{R}w_{i}^{r}\right]\\ ={𝔼}_{e_{i}^{1:R}}\left[{\left(\frac{w_{i}^{r}}{\sum_{r=1}^{R}w_{i}^{r}}\right)}^{2}\frac{\partial \log w_{i}^{r}}{\partial \theta_{i}^{r}}\frac{\partial \theta_{i}^{r}}{\partial \phi }\right]\\ \;\approx \frac{1}{S}\overset{S}{\underset{s=1}{\sum }}\overset{R}{\underset{r=1}{\sum }}{\left(\frac{w_{i}^{rs}}{\sum_{r=1}^{R}w_{i}^{rs}}\right)}^{2}\frac{\partial \log w_{i}^{rs}}{\partial \theta_{i}^{rs}}\frac{\partial \theta_{i}^{rs}}{\partial \phi }. \end{array}$$

Empirically, computing IW-ELBO and its gradient estimates can be numerically unstable due to exponential operations involved in $p\left(\theta_{i}^{rs}\right)$ and $q_{\phi }\left(\theta_{i}^{rs}|y_{i}\right)$. To solve this problem, we compute $v_{i}^{rs}=\log w_{i}^{rs}=\log p\left(y_{i}|\theta_{i}^{rs};M_{p}\right)-\log q_{\phi }\left(\theta_{i}^{rs}|y_{i}\right)+\log p\left(\theta_{i}^{rs}\right)$, and apply the well-known *log-sum-exp* trick (Zhang et al., 2021) to $\log\frac{1}{R}\sum w_{i}^{rs}$ in Equation (14) and $w_{i}^{rs}/\sum w_{i}^{rs}$ in Equations (15) and (16) as follows:

$$\begin{array}{l} \log\frac{1}{R}\overset{R}{\underset{r=1}{\sum }}w_{i}^{rs}=\underset{r}{\max}v_{i}^{rs}+\log\overset{R}{\underset{r=1}{\sum }}\exp\left(v_{i}^{rs}-\underset{r}{\max}v_{i}^{rs}\right)-\log R,\\ \frac{w_{i}^{rs}}{\sum_{r=1}^{R}w_{i}^{rs}}=\frac{\exp\left(v_{i}^{rs}-\max_{r}v_{i}^{rs}\right)}{\sum_{r=1}^{R}\exp\left(v_{i}^{rs}-\max_{r}v_{i}^{rs}\right)}. \end{array}$$

### 2.3. Implementation details

#### 2.3.1. MLP and optimization

We provide a basic overview of *multilayer perceptron* (MLP) applied in this study, which is used to model the variational distribution *q* as in Equation (8). For more details about MLP and DNN, we recommend readers to Goodfellow et al. (2016).

Multilayer perceptron, also known as *feedforward neural networks* (FNN), is one of the most popular architectures of neural networks because of its simple form and flexibility. To approximate an unknown function f^*^ such that ***u*** = f^*^(***v***) where ***v*** ∈ ℝ^*P*^, ***u*** ∈ ℝ^*Q*^, MLP takes the recursive form ***h***_*l*_ = *f*_*l*_(**W**_*l*_***h***_*l*−1_ + ***b***_*l*_), *l* = 1, …, *L*, and ***h***_0_ = ***v***, ***h***_*L*_ = ***u***. Here, *f*_1_, …, *f*_*L*_ are scalar functions which are almost everywhere differentiable and are applied elementwisely when inputs are vectors. These functions are typically termed as *activation* functions. When *f*_1_, …, *f*_*L*_ are set to identity function *g*(*z*) = *z*, MLP will reduce to linear regression; using non-linear activation functions, we get a flexible function *u* = *f*(*v*). MLP has been shown an universal approximator under a variety of activation functions (Cybenko, 1989; Hornik, 1991; Sonoda and Murata, 2017), including *sigmoid function* *g*(*x*) = 1/(*e*^−*x*^ + 1), *rectified linear unit function* (ReLU, Nair and Hinton, 2010) *g*(*x*) = max(0, *x*), and *hyperbolic tangent function* (Tanh) *g*(*x*) = (*e*^*x*^ − *e*^−*x*^)/(*e*^*x*^ + *e*^−*x*^); refer to Goodfellow et al. (2016) for other choices of activation functions. In this article, we use Tanh activation for *f*_1_…, *f*_*L*−1_.

The *f*_*L*_ at the last layer is chosen depending on the data form. To see this, note that the last layer of MLP ***u*** = *f*_*L*_(**W**_*L*_***h***_*L*−1_ + ***b***_*L*_) can be seen as a generalized linear model with independent variable ***h***_*L*−1_. When ***u*** is continuous, *f*_*L*_ can be set to the identity function and we get the last layer a linear regression. When ***u*** is binary (categorical), *f*_*L*_ can be set to the sigmoid (softmax) function and we get a logistic (multinomial logistic) regression, respectively.

In this article, we use the following encoder network

$$\begin{array}{l} h_{i}=\text{Tanh}\left(b_{L}+{\text{W}}_{L}\text{Tanh}\left(b_{L-1}+\ldots \text{Tanh}\left(b_{1}+{\text{W}}_{1}y_{i}\right)\right)\ldots \;\right),\\ \mu_{i}={\text{W}}_{\mu }h_{i}+b_{\mu },\\ \sigma_{i}^{2}=\exp\left({\text{W}}_{\sigma^{2}}h_{i}+b_{\sigma^{2}}\right). \end{array}$$

Here, ***h***_*i*_ denotes the intermediate output of the encoder given input the *i*-th individual data ***y***_*i*_, and we have $\phi =\left\{{\text{W}}_{1},b_{1},\ldots ,{\text{W}}_{L},b_{L},{\text{W}}_{\mu },b_{\mu },{\text{W}}_{\sigma^{2}},b_{\sigma^{2}}\right\}$. In the decoder, to effectively utilize the gradient based method, following Kucukelbir et al. (2017), we map ***c*** and ***d*** from constrained ranges [0, 1]^*J*^ to unconstrained space ℝ^*J*^ through the differentiable logit(*x*) = log *x*/(1 − *x*) transformation and conduct gradient ascent in the unconstrained space. To avoid cluttering, we still use original notations ***c*** and ***d*** in the following.

#### 2.3.2. Handling missing data

When ***y***_*i*_ given latent factors ***θ***_*i*_ are conditionally independent, exactly as the MIRT models assume, MLP based VAE and IWAE can handle incomplete data containing entries *missing at random* (MAR) readily (Nazabal et al., 2020). Here, we provide a brief summary of the *input drop-out* trick.

First, we replace missing entries in ***y***_*i*_ with zeros and denote the resultant vector as ${\tilde{y}}_{i}$; we further use indicator vector **1**_*i*_ to record which entries are observed, specifically, **1**_*ij*_ ≜ **1**(*y*_*ij*_ is observed)^1^. Next, we replace $w_{i}^{r}$ with ${\tilde{w}}_{i}^{r}=\exp{ṽ}_{i}^{r}$ where ${ṽ}_{i}^{r}$ is defined as

$$\begin{array}{l} {ṽ}_{i}^{r}=\overset{J}{\underset{j=1}{\sum }}\left[1_{ij}\log p\left({ỹ}_{ij}|{\tilde{\theta }}_{i}^{r};M_{p}\right)\right]-\log q_{\phi }\left({\tilde{\theta }}_{i}^{r}|{\tilde{y}}_{i}\right)+\log p\left({\tilde{\theta }}_{i}^{r}\right). \end{array}$$

For now, we use ${\tilde{\theta }}_{i}$ to emphasize that the inference network takes ${\tilde{y}}_{i}$ as input. Next we show the imputing missing entries with 0 does not influence the training. For *M*_*p*_, based on Equation (14), its gradient estimate is determined by $\nabla_{M_{p}}{ṽ}_{i}^{rs}$ and does not depend on imputed entries because of the multiplication of **1**_*ij*_, therefore *M*_*p*_ is also independent of them.

Additionally, if neither $q_{\phi }\left({\tilde{\theta }}_{i}|{\tilde{y}}_{i}\right)$ nor ${\tilde{\theta }}_{i}$ is affected by imputed entries, then such imputation will not influence the model training as $v_{i}^{r}$ (and $w_{i}^{r}$) does not rely on these entries. To this end, we rely on the MLP architecture. The output of each neuron in MLP is a non-linear transformation of a linear combination of its inputs. This property ensures that all intermediate states and output of the inference network, which determines ***μ***_*i*_ and ***σ***_*i*_ for variational distribution $q_{\phi }\left({\tilde{\theta }}_{i}|{\tilde{y}}_{i}\right)$, does not depend on zero entries in its inputs.

These observations together guarantee the condition for $v_{i}^{r}$ (and $w_{i}^{r}$) being independent of imputed entries, as in both ELBO and IW-ELBO, gradient estimates of all parameters are determined by collections of these terms.

#### 2.3.3. Training strategy and hyperparameter choices

We propose a three-stage training strategy for VAE by enhancing it with IW-ELBO. We first train a standard VAE through maximizing its own objective function ELBO. After reaching a local optimum, we train it to maximize the tighter IW-ELBO until it converges again. Since the computation cost of IW-ELBO is more expensive than ELBO, our strategy is cheaper than training an IWAE from scratch. We refer to our model as *importance-weighted sampling enhanced VAE*(IWVAE).

To be more specific, in the first 1% of total iterations, we apply the *KL annealing* technique, i.e., at step *t*, we multiply the KL divergence term *D*_KL_[*q*_ϕ_(***θ***_*i*_ ∣ ***y***_*i*_)||*p*(***θ***_*i*_)] by a factor $\frac{t}{T_{\text{anl}}}$, where *T*_anl_ = ⌈0.01*T*_max_⌉ and *T*_max_ = 2,00,000 is a pre-specified maximum number of iterations to avoid the algorithm running forever due to convergence issues. In this stage, the weight of the KL term increases from 0 to 1 linearly. KL annealing has shown great improvement in deep generative models (Gulrajani et al., 2016; Sønderby et al., 2016). The rationale behind this technique is that the KL divergence term can over-regularize the model by forcing the approximate posterior *q*_ϕ_(***θ***_*i*_) close to the prior *p*(***θ***_*i*_) and leading the model to converge early to unsatisfactory local minimums. To mitigate this issue, at the beginning of training, we simply reduce the effect of the KL term. During the annealing stage, we fix ***c*** and ***d*** and only update ϕ, **A**, ***b***.

After the annealing stage, we train IWVAE until its estimated ELBO converges such that the averaged ELBO value in every 100 steps stops increasing for *L* = 50 times. We refer to this stage as *ELBO converging*. Finally, we use importance-weighted samples to train IWVAE until it converges again in terms of IW-ELBO with this same rule. This stage is referred to *IW-ELBO converging*. After this stage, we end up training.

Algorithm 1 demonstrates our training method in a simplified version where at each step only 1 sample is drawn randomly from the data to estimate gradients. In practice, people can instead collect multiple samples (known as a *mini batch*) at each step and take the average for better gradients estimators. In practice, we used a mini batch size of 16 for each iteration step throughout all stages, *S* = 1 Monte Carlo sample in all three stages, and *R* = 5 importance samples in the last IW-ELBO converging stage following (Urban and Bauer, 2021). In terms of parameter updates, we use stochastic gradient ascent with fixed step size to maximize the ELBO or IW-ELBO. We assign a smaller step size (0.001) for parameters ***c*** and ***d*** as their ranges are smaller, and all other parameters are optimized with step size (0.01). No further tweaks such as gradient clippings (Pascanu et al., 2013) are used.

**Algorithm 1:** Stochastic gradient ascent of IWVAE.

**Input:** data **Y**; latent factor's dimension *K*; Monte Carlo and importance sample sizes *S, R*; maximum number of iterations *T*_max_.
Initialize $\hat{\phi },{\hat{M}}_{p}$ using random samples
**(KL annealing stage)**
**while** iteration number *t* not reaching *T*_anl_ = ⌈0.01*T*_max_⌉ **do**
randomly draw ***y***_*i*_ from *Y*;
draw *S* samples $\theta_{i}^{s}\sim N\left(\mu_{i},\sigma_{i}^{2}\right)$ with Equation (10) where $\left(\mu_{i},\sigma_{i}^{2}\right)=F_{\phi }\left(y_{i}\right)$;
compute $\log p\left(y_{i}|\theta_{i}^{s};M_{p}\right)$ with Equation (1) or Equation (2), *D*_KL_[*q*_ϕ_(***θ***_*i*_ ∣ ***y***_*i*_)||*p*(***θ***_*i*_)] with Equation (11). Take 1 gradient ascent step on
$$\begin{array}{l} \hat{\phi },{\hat{M}}_{p}=\underset{\phi ,M_{p}}{\mathrm{argmax}}\frac{1}{S}\overset{S}{\underset{s=1}{\sum }}\log p\left(y_{i}|\theta_{i}^{s};M_{p}\right)-\frac{t}{T_{\text{anl}}}D_{\mathrm{KL}}\left[q_{\phi }\left(\theta_{i}|y_{i}\right)||p\left(\theta_{i}\right)\right] \end{array}$$
**end while**
**(ELBO converging stage)**
**while** iteration number *t* not reaching *T*_max_ and ELBO not converging **do**
randomly draw ***y***_*i*_ from *Y*;
draw *S* samples $\theta_{i}^{s}\sim N\left(\mu_{i},\sigma_{i}^{2}\right)$ with Equation (10) where $\left(\mu_{i},\sigma_{i}^{2}\right)=F_{\phi }\left(y_{i}\right)$;
compute $\log p\left(y_{i}|\theta_{i}^{s};M_{p}\right)$ with Equation (1) or Equation (2), *D*_KL_[*q*_ϕ_(***θ***_*i*_ ∣ ***y***_*i*_)||*p*(***θ***_*i*_)] with Equation (11). Take 1 gradient ascent step on
$$\begin{array}{l} \hat{\phi },{\hat{M}}_{p}=\underset{\phi ,M_{p}}{\mathrm{argmax}}\frac{1}{S}\overset{S}{\underset{s=1}{\sum }}\log p\left(y_{i}|\theta_{i}^{s};M_{p}\right)-D_{\mathrm{KL}}\left[q_{\phi }\left(\theta_{i}|y_{i}\right)||p\left(\theta_{i}\right)\right] \end{array}$$
**end while**
**(IW-ELBO converging stage)**
**while** iteration number *t* not reaching *T*_max_ and IW-ELBO not converging **do**
randomly draw ***y***_*i*_ from *Y*;
draw *SR* samples $\theta_{i}^{rs}\sim N\left(\mu_{i},\sigma_{i}^{2}\right)$ with Equation (10) where $\left(\mu_{i},\sigma_{i}^{2}\right)=F_{\phi }\left(y_{i}\right)$;
compute $\log p\left(y_{i}|\theta_{i}^{s};M_{p}\right)$ with Equation (1) or Equation (2), $w_{i}^{rs}=\exp\left[\log p\left(y_{i}|\theta_{i}^{rs};M_{p}\right)-\log q_{\phi }\left(\theta_{i}^{rs}|y_{i}\right)+\log p\left(\theta_{i}^{rs}\right)\right]$. Take 1 gradient ascent step on
$$\begin{array}{l} \hat{\phi },{\hat{M}}_{p}=\underset{\phi ,M_{p}}{\mathrm{argmax}}\frac{1}{S}\overset{S}{\underset{s=1}{\sum }}\left[\log\frac{1}{R}\overset{R}{\underset{r=1}{\sum }}w_{i}^{rs}\right] \end{array}$$
**end while**
**Output:** parameter estimates $\hat{\phi },{\hat{M}}_{p}$

## 3. Simulation study

### 3.1. Data generation

To evaluate the performances of applying IWVAE to M3PL and M4PL models, we conducted a thorough simulation study. We considered both within item and between item multidimensionality. In particular, for the within item multidimensionality, each item was loaded on two factors; and for the between item multidimensionality, each item was loaded on one factor. Under both settings, items dependency were distributed to different factors evenly in an indirect way through a sparse *J* × *K* loading matrix **A**. Specifically, we first generated a blocked diagonal submatrix **A**′. Next, we repeated two steps iteratively: (1) flipped **A**′ horizontally, and (2) concatenated to previous results, until we have the full s-shaped matrix **A**. When *J* is not a multiple of row numbers of **A**′, we truncated the resultant matrix at the bottom. To make the design more realistic and challenging, we considered a missing data design. For datasets with large *J*, it is impractical to have all items observed from every single respondent in realistic scenarios. To reflect this concern, we randomly masked a large portion (80% in our experiments) of responses from each respondent, assuming each respondent only answer 20% of the items.

Parameters *M*_*p*_ and latent factors Θ were generated as follows. For latent factor ***θ***_*i*_, under the *independent* factors setting, it was sampled from the standard multivariate Gaussian N(0,I). Under the *correlated* factors setting, a covariance matrix Σ was first generated and shared by all ***θ***_*i*_. Specifically, the diagonal entries were set to 1 so that each factor has unit variance; and off-diagonal (specifically, upper diagonal) entries were sampled independently from U(0,1). This Σ was accepted if it was positive semi-definitive, otherwise, another matrix was regenerated. For free parameters in the discrimination matrix $\alpha_{ij}\in {\text{A}}^{\prime }$, we sampled it from U(0.5,1.5). For *J* pairs of guessing and upper asymptote parameters (*c*_*j*_, *d*_*j*_), we sampled them from *c*_*j*_ ~ *Beta*(1, 9), *d*_*j*_ ~ *Beta*(9, 1) in parallel and kept them if all *c*_*j*_ < *d*_*j*_.

Our experiments were conducted as follows. First, we chose latent factors ***θ***_*i*_ for *i* = 1, …, *N* to be uncorrelated and studied two asymptotic regimes. Specifically, in the **single** asymptotic regime, the dimensions of items *J* and factors *K* were fixed to 100 and 5 respectively, and sample size *N* was increased from 500 to 10,000. In the **double** asymptotic regime, only *K* was fixed to 5 and *J* was increased from 100 to 500 as *N* grew. Under both settings, we chose *N* ∈ {500, 1,000, 5,000, 10,000} and in the double asymptotic settings we further chose *J* ∈ {100, 200, 300, 500}. Under each combination of *N, J, K*, we evaluated performances of IWVAE, MCEM, and MHRM on the M3(4)PL model by checking item parameters *M*_*p*_ estimation. Finally, we duplicated this series of experiments to correlated factors settings.

We implemented IWVAE in PyTorch (Paszke et al., 2019) and MCEM in the mirt R package (Chalmers, 2012). All experiments were run on the same *high performance computing cluster* (HPCC) with 4 CPUs and 4 GB memory, and no GPU was used. MHRM was implemented with FlexMIRT (Chung and Houts, 2020) and all experiments were fitted on a laptop with Intel Intel(R) Core(TM) i7-10750H CPU and 16 GB memory^2^. Because of the platform difference, we ran *B* = 100 independent replications for IWVAE and MCEM on each simulated dataset, and *B* = 20 replications for MHRM.

To evaluate the performances of MCEM, MHRM, and IWVAE, we followed Cho et al. (2021) and Urban and Bauer (2021) and reported *rooted mean squared error* (RMSE) across *B* independent experiment replications. Specifically, for each scalar parameter ξ (one of α_*jk*_, *b*_*j*_, *c*_*j*_, *d*_*j*_ for *j* = 1, …, *J, k* = 1, …, *K*), RMSE for each parameter was computed by

(17) $$\begin{array}{l} \text{RMSE}\left(\hat{\xi }\right)=\sqrt{\frac{1}{B}\overset{B}{\underset{b=1}{\sum }}{\left({\hat{\xi }}_{b}-\xi \right)}^{2}}, \end{array}$$

where ${\hat{\xi }}_{b}$ is the estimated value from the *b*-th replication. The final reported RMSEs were averages of corresponding entries in matrix **A** or vectors ***b***, ***c***, ***d***, and standard error were shown after each value in the parenthesis.

Note that the matrix **A** in MIRT (IRT) models can be only identified up to a rotation if no further prior constraint is imposed, and we conducted *post-hoc* processing on $\hat{\text{A}}$ following other literature. Our transformation consisted of three steps. First, we applied the *promax* (Hendrickson and White, 1964) rotation to the estimated $\hat{\text{A}}$, which allowed different factors to be correlated; we denoted this intermediate result with ${\hat{\text{A}}}^{r}$. Next, for each column in ${\hat{\text{A}}}^{r}$ that had a negative sum, we flipped its sign and the corresponding factor (refer to, e.g., Urban and Bauer, 2021), we marked the resultant matrix in this step as ${\hat{\text{A}}}^{rf}$. Finally, we searched over the best permutation of columns of ${\hat{\text{A}}}^{rf}$ such that RMSE was minimized, and the corresponding RMSEs were reported in tables.

We also utilized the CF-Quartimax rotation as in Cho et al. (2022) to evaluate the sparsity structure estimation of different methods. However, since sparsity estimation is not the main focus of this article, we defer presenting these results to the appendix.

Finally, Considering that M3PL is notoriously hard for MHRM to fit (Cho et al., 2021), and M4PL is expected to be more difficult, we reported the *success rate* of each method, which refers to the percentage of successful replications. The exact definition of *success* for different methods differs. For MCEM, it refers to the case where the MCEM algorithm terminates and provides estimates successfully, regardless of convergence^3^. For IWVAE, it also refers to successful termination without reaching the maximum iteration number, which implies proper convergence. The difference in *success*, as we shall see later, is influential: MHRM usually performed the best if it succeeds. MCEM, on the contrary, had much worse performances while *succeeding* in all experiments.

### 3.2. Numeric results

In this section, we show detailed numeric results on *M*_*p*_ estimations, which are summarized in Tables 1–8. In a nutshell, IWVAE achieved competitive or better performances compared to the two other statistical methods. IWVAE achieved much lower RMSE on nearly all item parameters in almost all experiments than MCEM; and unlike MHRM, IWVAE succeed in all experiments from small- to large-scale datasets. Additionally, IWVAE required much more scalable training times on all experiments, while MCEM and MHRM had time costs growing faster as sample size increased.

**Table 1:** Mean and SE of RMSE of *M*_*p*_ estimate on M4PL models under **single** regime setting, best results are in bold.

**N, J**	**Item structure**	**Model**	**rot(A)**	***b***	***c***	***d***	**Success rates**
500 100	Between	MCEM	9.400 ± 0.181	11.477 ± 0.424	0.175 ± 0.010	0.183 ± 0.010	1.00
		MHRM	/	/	/	/	/
		IWVAE	**0.674 ± 0.02**	**0.384 ± 0.027**	**0.081 ± 0.008**	**0.087 ± 0.008**	1.00
	Within	MCEM	10.406 ± 0.240	11.500 ± 0.481	0.163 ± 0.010	0.146 ± 0.008	1.00
		MHRM	/	/	/	/	/
		IWVAE	**0.744 ± 0.022**	**0.402 ± 0.034**	**0.073 ± 0.008**	**0.088 ± 0.008**	1.00
1000 100	Between	MCEM	8.230 ± 0.170	11.785 ± 0.450	0.189 ± 0.012	0.178 ± 0.011	1.00
		MHRM	/	/	/	/	/
		IWVAE	**0.498 ± 0.019**	**0.341 ± 0.028**	**0.079 ± 0.008**	**0.080 ± 0.008**	1.00
	Within	MCEM	7.799 ± 0.230	8.999 ± 0.464	0.132 ± 0.010	0.161 ± 0.011	1.00
		MHRM	/	/	/	/	/
		IWVAE	**0.609 ± 0.022**	**0.386 ± 0.029**	**0.069 ± 0.007**	**0.078 ± 0.008**	1.00
5,000 100	Between	MCEM	3.240 ± 0.156	4.351 ± 0.276	0.189 ± 0.011	0.155 ± 0.011	1.00
		MHRM	/	/	/	/	/
		IWVAE	**0.369 ± 0.027**	**0.378 ± 0.043**	**0.091 ± 0.011**	**0.082 ± 0.009**	1.00
	Within	MCEM	3.235 ± 0.221	2.939 ± 0.254	0.139 ± 0.012	0.133 ± 0.010	1.00
		MHRM	/	/	/	/	/
		IWVAE	**0.535 ± 0.029**	**0.383 ± 0.035**	**0.075 ± 0.008**	**0.086 ± 0.010**	1.00
10,000 100	Between	MCEM	1.988 ± 0.099	2.690 ± 0.184	0.174 ± 0.010	0.186 ± 0.011	1.00
		MHRM	/	/	/	/	/
		IWVAE	**0.379 ± 0.028**	**0.399 ± 0.042**	**0.084 ± 0.008**	**0.079 ± 0.008**	1.00
	Within	MCEM	1.823 ± 0.145	1.674 ± 0.151	0.136 ± 0.009	0.125 ± 0.007	1.00
		MHRM	/	/	/	/	/
		IWVAE	**0.516 ± 0.030**	**0.343 ± 0.038**	**0.084 ± 0.010**	**0.079 ± 0.008**	1.00

Factors are diagonal. **Between** item structure: each item depends on 1 factor. **Within** item structure: each item depends on 2 factors.

**Table 2:** Mean and SE of RMSE of *M*_*p*_ estimate on M4PL models under **double** regime setting, best results are in bold.

**N, J**	**Item structure**	**Model**	**rot(A)**	***b***	***c***	***d***	**Success rates**
500 100	Between	MCEM	9.400 ± 0.181	11.477 ± 0.424	0.175 ± 0.010	0.183 ± 0.010	1.00
		MHRM	/	/	/	/	/
		IWVAE	**0.674 ± 0.021**	**0.384 ± 0.027**	**0.081 ± 0.008**	**0.087 ± 0.008**	1.00
	Within	MCEM	10.406 ± 0.240	11.500 ± 0.481	0.163 ± 0.010	0.146 ± 0.008	1.00
		MHRM	/	/	/	/	/
		IWVAE	**0.744 ± 0.022**	**0.402 ± 0.034**	**0.073 ± 0.008**	**0.088 ± 0.008**	1.00
1000 200	Between	MCEM	5.397 ± 0.069	8.312 ± 0.188	0.180 ± 0.007	0.178 ± 0.007	1.00
		MHRM	/	/	/	/	/
		IWVAE	**0.500 ± 0.016**	**0.377 ± 0.025**	**0.080 ± 0.006**	**0.088 ± 0.007**	1.00
	Within	MCEM	8.242 ± 0.174	9.254 ± 0.354	0.151 ± 0.007	0.158 ± 0.007	1.00
		MHRM	/	/	/	/	/
		IWVAE	**0.600 ± 0.017**	**0.413 ± 0.024**	**0.080 ± 0.005**	**0.088 ± 0.007**	1.00
5,000 300	Between	MCEM	1.624 ± 0.058	1.519 ± 0.068	0.163 ± 0.006	0.156 ± 0.005	1.00
		MHRM	/	/	/	/	/
		IWVAE	**0.429 ± 0.014**	**0.338 ± 0.019**	**0.084 ± 0.005**	**0.081 ± 0.005**	1.00
	Within	MCEM	1.388 ± 0.079	0.876 ± 0.076	0.092 ± 0.005	0.086 ± 0.004	1.00
		MHRM	/	/	/	/	/
		IWVAE	**0.564 ± 0.015**	**0.319 ± 0.019**	**0.083 ± 0.005**	**0.080 ± 0.005**	1.00
10,000 500	Between	MCEM	1.022 ± 0.023	1.152 ± 0.036	0.150 ± 0.004	0.153 ± 0.004	1.00
		MHRM	/	/	/	/	/
		IWVAE	**0.432 ± 0.012**	**0.338 ± 0.015**	**0.086 ± 0.004**	**0.086 ± 0.004**	1.00
	Within	MCEM	0.930 ± 0.018	0.993 ± 0.031	0.119 ± 0.004	0.119 ± 0.003	1.00
		MHRM	/	/	/	/	/
		IWVAE	**0.564 ± 0.013**	**0.346 ± 0.015**	**0.085 ± 0.004**	**0.087 ± 0.004**	1.00

Factors are diagonal. **Between** item structure: each item depends on 1 factor. **Within** item structure: each item depends on 2 factors.

**Table 3:** Mean and SE of RMSE of *M*_*p*_ estimate on M3PL models under **single** regime setting, best results are in bold.

**N, J**	**Item structure**	**Model**	**rot(A)**	***b***	***c***	**Success rates**
500 100	Between	MCEM	10.020 ± 0.318	13.638 ± 0.752	0.213 ± 0.013	1.00
		MHRM	**0.217 ± 0.026**	0.567 ± 0.080	0.099 ± 0.007	0.35
		IWVAE	0.641 ± 0.022	**0.391 ± 0.031**	**0.081 ± 0.008**	1.00
	Within	MCEM	8.133 ± 0.459	8.687 ± 0.727	0.194 ± 0.014	1.00
		MHRM	**0.417 ± 0.034**	**0.345 ± 0.049**	0.078 ± 0.005	0.40
		IWVAE	0.708 ± 0.021	0.461 ± 0.039	**0.073 ± 0.008**	1.00
1000 100	Between	MCEM	5.338 ± 0.287	7.799 ± 0.544	0.237 ± 0.016	1.00
		MHRM	**0.159 ± 0.017**	**0.280 ± 0.025**	0.089 ± 0.007	0.30
		IWVAE	0.492 ± 0.019	0.320 ± 0.026	**0.079 ± 0.008**	1.00
	Within	MCEM	2.564 ± 0.235	3.023 ± 0.423	0.120 ± 0.011	1.00
		MHRM	**0.438 ± 0.038**	**0.313 ± 0.040**	0.075 ± 0.005	0.65
		IWVAE	0.590 ± 0.020	0.325 ± 0.024	**0.069 ± 0.007**	1.00
5,000 100	Between	MCEM	1.031 ± 0.110	1.190 ± 0.204	0.144 ± 0.013	1.00
		MHRM	**0.151 ± 0.024**	0.264 ± 0.028	**0.090 ± 0.008**	0.30
		IWVAE	0.403 ± 0.024	**0.259 ± 0.028**	0.091 ± 0.011	1.00
	Within	MCEM	0.881 ± 0.063	0.575 ± 0.077	0.097 ± 0.009	1.00
		MHRM	**0.292 ± 0.035**	**0.123 ± 0.009**	**0.033 ± 0.004**	0.90
		IWVAE	0.562 ± 0.026	0.279 ± 0.032	0.075 ± 0.008	1.00
10,000 100	Between	MCEM	0.810 ± 0.078	1.008 ± 0.169	0.112 ± 0.011	1.00
		MHRM	**0.106 ± 0.019**	0.381 ± 0.131	**0.055 ± 0.006**	0.70
		IWVAE	0.393 ± 0.027	**0.318 ± 0.036**	0.084 ± 0.008	1.00
	Within	MCEM	0.754 ± 0.045	0.662 ± 0.129	0.076 ± 0.007	1.00
		MHRM	**0.343 ± 0.035**	**0.154 ± 0.012**	**0.040 ± 0.004**	0.75
		IWVAE	0.535 ± 0.027	0.283 ± 0.039	0.084 ± 0.010	1.00

Factors are diagonal. **Between** item structure: each item depends on 1 factor. **Within** item structure: each item depends on 2 factors.

Tables 1–4 show RMSE of *M*_*p*_ estimation in M4PL and M3PL models under single and double asymptotic regimes, where different entries in each latent factor ***θ*** were generated independently. Two item structures were reported together in the same table. First, we observed that MCEM and IWVAE are more robust, as they succeed in all experiments, while MHRM achieved a success rate of 50% on few experiments in the M3PL model. Next, IWVAE reached much lower RMSE than MCEM, especially on small to medium sized data. In addition, IWVAE showed similar tendencies as MCEM and MHRM did: as *N* grew, its RMSE showed remarkable decreases, and on more challenging within-item structure scenarios, IWVAE also had slightly higher RMSEs.

**Table 4:** Mean and SE of RMSE of *M*_*p*_ estimate on M3PL models under **double** regime setting, best results are in bold.

**N, J**	**Item structure**	**Model**	**rot(A)**	***b***	***c***	**Success rates**
500 100	Between	MCEM	10.020 ± 0.318	13.638 ± 0.752	0.213 ± 0.013	1.00
		MHRM	**0.217 ± 0.026**	0.567 ± 0.080	0.099 ± 0.007	0.35
		IWVAE	0.641 ± 0.022	**0.391 ± 0.031**	**0.081 ± 0.008**	1.00
	Within	MCEM	8.133 ± 0.459	8.687 ± 0.727	0.194 ± 0.014	1.00
		MHRM	**0.417 ± 0.034**	**0.345 ± 0.049**	0.078 ± 0.005	0.40
		IWVAE	0.708 ± 0.021	0.461 ± 0.039	**0.073 ± 0.008**	1.00
1000 200	Between	MCEM	5.224 ± 0.192	8.544 ± 0.407	0.243 ± 0.011	1.00
		MHRM	/	/	/	0.00
		IWVAE	**0.506 ± 0.015**	**0.348 ± 0.024**	**0.080 ± 0.006**	1.00
	Within	MCEM	2.976 ± 0.193	3.441 ± 0.304	0.157 ± 0.008	1.00
		MHRM	/	/	/	0.00
		IWVAE	**0.638 ± 0.015**	**0.345 ± 0.020**	**0.080 ± 0.005**	1.00
5,000 300	Between	MCEM	0.612 ± 0.019	0.508 ± 0.048	0.114 ± 0.007	1.00
		MHRM	/	/	/	0.00
		IWVAE	**0.459 ± 0.012**	**0.261 ± 0.016**	**0.084 ± 0.005**	1.00
	Within	MCEM	0.693 ± 0.015	0.306 ± 0.020	**0.075 ± 0.004**	1.00
		MHRM	/	/	/	0.00
		IWVAE	**0.595 ± 0.013**	**0.271 ± 0.017**	0.082 ± 0.005	1.00
10,000 500	Between	MCEM	0.561 ± 0.010	0.572 ± 0.032	0.097 ± 0.004	1.00
		MHRM	/	/	/	0.00
		IWVAE	**0.465 ± 0.011**	**0.258 ± 0.013**	**0.086 ± 0.004**	1.00
	Within	MCEM	0.678 ± 0.010	0.581 ± 0.047	**0.058 ± 0.003**	1.00
		MHRM	/	/	/	0.00
		IWVAE	**0.592 ± 0.011**	**0.271 ± 0.014**	0.085 ± 0.004	1.00

Factors are diagonal. **Between** item structure: each item depends on 1 factor. **Within** item structure: each item depends on 2 factors.

For experiments where each latent factor ***θ*** has correlated components, we organized results in the same way as before. Tables 5–8 show RMSE of *M*_*p*_ estimation in M4PL and M3PL models under single and double asymptotic regimes. Again, we observed similar results from IWVAE to MCEM and MHRM in terms of success times and RMSE, indicating the advantage of the proposed IWVAE method.

**Table 5:** Mean and SE of RMSE of *M*_*p*_ estimate on M4PL models under **single** regime setting, best results are in bold.

**N, J**	**Item structure**	**Model**	**rot(A)**	***b***	***c***	***d***	**Success rates**
500 100	Between	MCEM	11.248 ± 0.217	13.315 ± 0.491	0.172 ± 0.011	0.178 ± 0.010	1.00
		MHRM	/	/	/	/	/
		IWVAE	**0.654 ± 0.023**	**0.363 ± 0.024**	**0.081 ± 0.008**	**0.087 ± 0.008**	1.00
	Within	MCEM	12.038 ± 0.286	12.611 ± 0.665	0.148 ± 0.010	0.138 ± 0.008	1.00
		MHRM	/	/	/	/	/
		IWVAE	**0.736 ± 0.022**	**0.416 ± 0.032**	**0.073 ± 0.008**	**0.088 ± 0.008**	1.00
1000 100	Between	MCEM	8.231 ± 0.159	11.774 ± 0.417	0.180 ± 0.011	0.189 ± 0.012	1.00
		MHRM	/	/	/	/	/
		IWVAE	**0.486 ± 0.019**	**0.334 ± 0.028**	**0.079 ± 0.008**	**0.080 ± 0.008**	1.00
	Within	MCEM	7.181 ± 0.302	7.160 ± 0.527	0.108 ± 0.009	0.134 ± 0.010	1.00
		MHRM	/	/	/	/	/
		IWVAE	**0.623 ± 0.020**	**0.408 ± 0.033**	**0.069 ± 0.007**	**0.078 ± 0.008**	1.00
5,000 100	Between	MCEM	3.315 ± 0.167	4.790 ± 0.354	0.182 ± 0.014	0.159 ± 0.012	1.00
		MHRM	/	/	/	/	/
		IWVAE	**0.379 ± 0.026**	**0.363 ± 0.043**	**0.091 ± 0.011**	**0.082 ± 0.009**	1.00
	Within	MCEM	1.886 ± 0.152	1.468 ± 0.186	0.094 ± 0.009	0.087 ± 0.007	1.00
		MHRM	/	/	/	/	/
		IWVAE	**0.529 ± 0.031**	**0.397 ± 0.037**	**0.075 ± 0.008**	**0.086 ± 0.010**	1.00
10,000 100	Between	MCEM	1.918 ± 0.114	2.555 ± 0.213	0.157 ± 0.010	0.176 ± 0.011	1.00
		MHRM	/	/	/	/	/
		IWVAE	**0.380 ± 0.028**	**0.402 ± 0.040**	**0.084 ± 0.008**	**0.079 ± 0.008**	1.00
	Within	MCEM	1.127 ± 0.075	0.943 ± 0.079	0.095 ± 0.007	0.087 ± 0.006	1.00
		MHRM	/	/	/	/	/
		IWVAE	**0.520 ± 0.033**	**0.360 ± 0.039**	**0.085 ± 0.010**	**0.079 ± 0.008**	1.00

Factors are correlated. **Between** item structure: each item depends on 1 factor. **Within** item structure: each item depends on 2 factors.

**Table 6:** Mean and SE of RMSE of *M*_*p*_ estimate on M4PL models under **double** regime setting, best results are in bold.

**N, J**	**Item structure**	**Model**	**rot(A)**	***b***	***c***	***d***	**Success rates**
500 100	Between	MCEM	11.248 ± 0.217	13.315 ± 0.491	0.172 ± 0.011	0.178 ± 0.010	1.00
		MHRM	/	/	/	/	/
		IWVAE	**0.654 ± 0.023**	**0.363 ± 0.024**	**0.081 ± 0.008**	**0.087 ± 0.008**	1.00
	Within	MCEM	12.038 ± 0.286	12.611 ± 0.665	0.148 ± 0.010	0.138 ± 0.008	1.00
		MHRM	/	/	/	/	/
		IWVAE	**0.736 ± 0.022**	**0.416 ± 0.032**	**0.073 ± 0.008**	**0.088 ± 0.008**	1.00
1000 200	Between	MCEM	8.314 ± 0.097	11.742 ± 0.298	0.178 ± 0.008	0.197 ± 0.008	1.00
		MHRM	/	/	/	/	/
		IWVAE	**0.503 ± 0.015**	**0.362 ± 0.024**	**0.080 ± 0.006**	**0.088 ± 0.007**	1.00
	Within	MCEM	7.323 ± 0.213	7.628 ± 0.362	0.125 ± 0.006	0.131 ± 0.007	1.00
		MHRM	/	/	/	/	/
		IWVAE	**0.609 ± 0.015**	**0.407 ± 0.024**	**0.080 ± 0.005**	**0.088 ± 0.007**	1.00
5,000 300	Between	MCEM	2.041 ± 0.090	2.292 ± 0.154	0.143 ± 0.006	0.139 ± 0.006	1.00
		MHRM	/	/	/	/	/
		IWVAE	**0.426 ± 0.013**	**0.340 ± 0.020**	**0.084 ± 0.005**	**0.081 ± 0.005**	1.00
	Within	MCEM	1.049 ± 0.053	0.525 ± 0.065	**0.066 ± 0.005**	**0.059 ± 0.004**	1.00
		MHRM	/	/	/	/	/
		IWVAE	**0.582 ± 0.014**	**0.339 ± 0.019**	0.083 ± 0.005	0.080 ± 0.005	1.00
10,000 500	Between	MCEM	1.062 ± 0.036	1.163 ± 0.060	0.129 ± 0.004	0.131 ± 0.004	1.00
		MHRM	/	/	/	/	/
		IWVAE	**0.426 ± 0.011**	**0.332 ± 0.015**	**0.086 ± 0.004**	**0.086 ± 0.004**	1.00
	Within	MCEM	0.884 ± 0.015	0.944 ± 0.031	0.098 ± 0.003	0.099 ± 0.003	1.00
		MHRM	/	/	/	/	/
		IWVAE	**0.562 ± 0.012**	**0.367 ± 0.016**	**0.085 ± 0.004**	**0.087 ± 0.004**	1.00

Factors are correlated. **Between** item structure: each item depends on 1 factor. **Within** item structure: each item depends on 2 factors.

**Table 7:** Mean and SE of RMSE of *M*_*p*_ estimate on M3PL models under **single** regime setting, best results are in bold.

**N, J**	**Item structure**	**Model**	**rot(A)**	***b***	***c***	**Success rates**
500 100	Between	MCEM	9.137 ± 0.396	12.034 ± 0.799	0.192 ± 0.012	1.00
		MHRM	**0.331 ± 0.040**	0.441 ± 0.062	**0.077 ± 0.006**	0.05
		IWVAE	0.659 ± 0.020	**0.411 ± 0.029**	0.081 ± 0.008	1.00
	Within	MCEM	7.644 ± 0.465	7.291 ± 0.612	0.153 ± 0.011	1.00
		MHRM	**0.492 ± 0.048**	**0.364 ± 0.054**	**0.064 ± 0.005**	0.45
		IWVAE	0.733 ± 0.020	0.440 ± 0.038	0.073 ± 0.008	1.00
1000 100	Between	MCEM	5.231 ± 0.279	8.080 ± 0.573	0.219 ± 0.014	1.00
		MHRM	**0.284 ± 0.027**	0.350 ± 0.041	0.090 ± 0.007	0.25
		IWVAE	0.483 ± 0.019	**0.312 ± 0.024**	**0.079 ± 0.008**	1.00
	Within	MCEM	2.855 ± 0.299	2.912 ± 0.457	0.118 ± 0.010	1.00
		MHRM	**0.428 ± 0.034**	0.336 ± 0.020	**0.045 ± 0.004**	0.80
		IWVAE	0.601 ± 0.024	**0.299 ± 0.026**	0.069 ± 0.007	1.00
5,000 100	Between	MCEM	0.762 ± 0.063	0.638 ± 0.117	0.130 ± 0.015	1.00
		MHRM	**0.242 ± 0.019**	**0.120 ± 0.011**	**0.051 ± 0.005**	0.75
		IWVAE	0.415 ± 0.023	0.256 ± 0.031	0.091 ± 0.011	1.00
	Within	MCEM	0.986 ± 0.075	0.421 ± 0.054	0.066 ± 0.006	1.00
		MHRM	**0.357 ± 0.034**	0.460 ± 0.013	**0.030 ± 0.002**	0.80
		IWVAE	0.578 ± 0.029	**0.308 ± 0.034**	0.075 ± 0.008	1.00
10,000 100	Between	MCEM	0.917 ± 0.107	1.073 ± 0.170	0.110 ± 0.012	1.00
		MHRM	**0.155 ± 0.013**	**0.114 ± 0.014**	**0.037 ± 0.006**	0.65
		IWVAE	0.397 ± 0.027	0.297 ± 0.035	0.084 ± 0.008	1.00
	Within	MCEM	0.898 ± 0.056	0.751 ± 0.165	0.057 ± 0.006	1.00
		MHRM	**0.378 ± 0.036**	0.461 ± 0.027	**0.017 ± 0.002**	0.40
		IWVAE	0.547 ± 0.032	**0.302 ± 0.040**	0.084 ± 0.010	1.00

Factors are correlated. **Between** item structure: each item depends on 1 factor. **Within** item structure: each item depends on 2 factors.

**Table 8:** Mean and SE of RMSE of *M*_*p*_ estimate on M3PL models under **double** regime setting, best results are in bold.

**N, J**	**Item structure**	**Model**	**rot(A)**	***b***	***c***	**Success rates**
500 100	Between	MCEM	9.137 ± 0.396	12.034 ± 0.799	0.192 ± 0.012	1.00
		MHRM	**0.331 ± 0.040**	0.441 ± 0.062	**0.077 ± 0.006**	0.05
		IWVAE	0.659 ± 0.020	**0.411 ± 0.029**	0.081 ± 0.008	1.00
	Within	MCEM	7.644 ± 0.465	7.291 ± 0.612	0.153 ± 0.011	1.00
		MHRM	**0.492 ± 0.048**	**0.364 ± 0.054**	**0.064 ± 0.005**	0.45
		IWVAE	0.733 ± 0.020	0.440 ± 0.038	0.073 ± 0.008	1.00
1000 200	Between	MCEM	4.662 ± 0.200	7.364 ± 0.410	0.220 ± 0.010	1.00
		MHRM	/	/	/	0.00
		IWVAE	**0.512 ± 0.014**	**0.329 ± 0.022**	**0.080 ± 0.006**	1.00
	Within	MCEM	2.233 ± 0.171	2.294 ± 0.266	0.104 ± 0.006	1.00
		MHRM	/	/	/	0.00
		IWVAE	**0.670 ± 0.014**	**0.318 ± 0.020**	**0.080 ± 0.005**	1.00
5,000 300	Between	MCEM	0.576 ± 0.017	0.450 ± 0.048	0.101 ± 0.006	1.00
		MHRM	/	/	/	0.00
		IWVAE	**0.450 ± 0.012**	**0.263 ± 0.016**	**0.084 ± 0.005**	1.00
	Within	MCEM	0.728 ± 0.013	**0.215 ± 0.017**	**0.050 ± 0.003**	1.00
		MHRM	/	/	/	0.00
		IWVAE	**0.624 ± 0.013**	0.290 ± 0.018	0.082 ± 0.005	1.00
10,000 500	Between	MCEM	0.571 ± 0.012	0.720 ± 0.049	0.089 ± 0.004	1.00
		MHRM	/	/	/	0.00
		IWVAE	**0.451 ± 0.011**	**0.246 ± 0.013**	**0.086 ± 0.004**	1.00
	Within	MCEM	0.858 ± 0.018	1.411 ± 0.087	**0.065 ± 0.002**	1.00
		MHRM	/	/	/	0.00
		IWVAE	**0.587 ± 0.011**	**0.267 ± 0.014**	0.085 ± 0.004	1.00

Factors are correlated. **Between** item structure: each item depends on 1 factor. **Within** item structure: each item depends on 2 factors.

**Figure 1:**
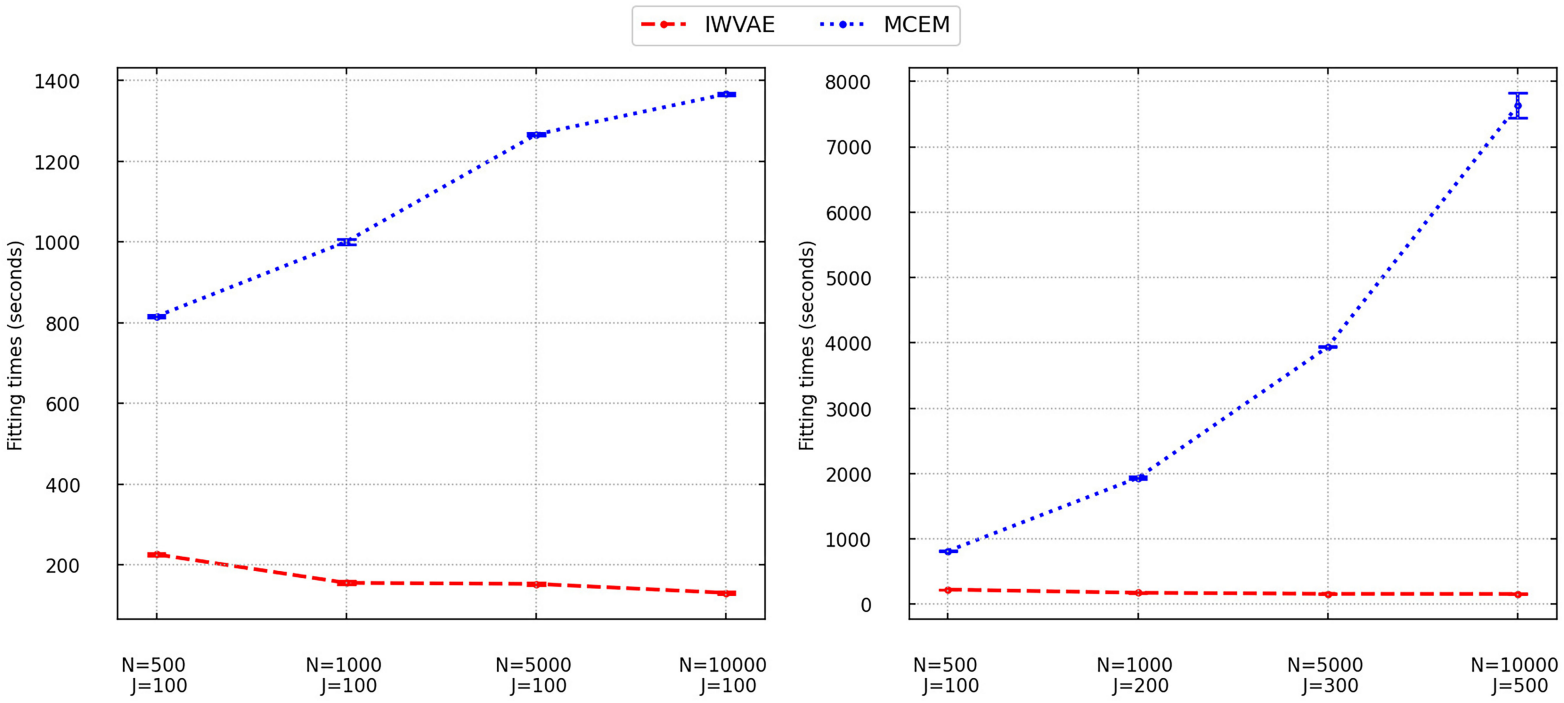
Fitting times for IWVAE and MCEM with M3PL model under the single (different sample sizes *N* and fixed item dimension *J*) and double [different (*N, J*)] asymptotic regimes. Vertical bar areas mark empirical 95% intervals.

**Figure 2:**
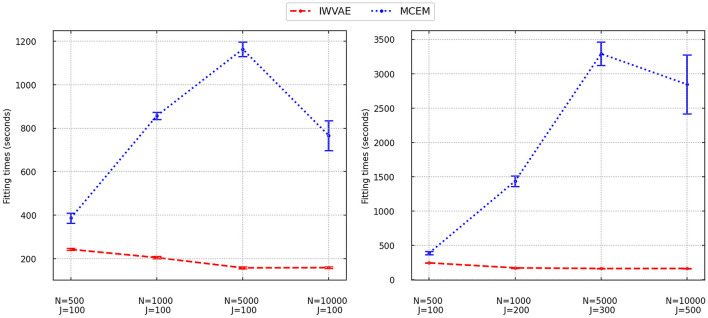
Fitting times for IWVAE and MCEM with M4PL model under the single (different sample sizes *N* and fixed item dimension *J*) and double [different (*N, J*)] asymptotic regimes. Vertical bar areas mark empirical 95% intervals.

We finally analyzed the fitting time of IWVAE and MCEM and reported averaged time with stand error (in shadow) in Figure 1 (M3PL) and Figure 2 (M4PL). We combined different factor settings (*independent* and *correlated*), and item structures (*between* and *within*) for every pair of sample size *N* and item size *J*. Each point contains 80 trials. As MHRM could not fit M4PL and its convergence results under M3PL were not stable, here, we do not report their results. From Figures 1, 2, compared to MCEM, IWVAE required significantly lower fitting time. Unlike MCEM, IWVAE had a much more stable fitting time across different data sizes, which was also observed in Urban and Bauer (2021) for estimating M2PL. As in Urban and Bauer (2021), we also note that the computational time of IWVAE appeared not to increase with *N* and *J*, which may be due to that VAE-based models are more difficult to train on small data sets. Similarly, in some cases, the computational time of MCEM also dropped when *N* increased to 10, 000, which may also be because of the easier convergence of the algorithm for the larger datasets. Moreover, we observed that fitting time of MCEM under M4PL depended more on the choices of initialization, revealed by the width of empirical intervals in Figure 2.

## 4. Real data analysis

In this section, we evaluated the performance of IWVAE, MCEM, and MHRM on the multistage testing (MST) dataset from the National Assessment of Education Progress (NAEP). The data is from the 2011 grade 8 math assessment study. The NAEP MST design takes a two-stage form: in the routing stage, a block of items with medium difficulty is administered. Then in the second stage, there are three targeted blocks with varying difficulty—blocks of easy, medium, and hard items. Based on a person's performance in the routing block, one of the three targeted blocks is assigned in the second-stage accordingly. Because the assignment in stage II depends on the observed student performance in stage I, the MST design essentially generates a unique missing-at-random pattern. Due to the prevalence of MST design in large scale assessments, it would interesting to evaluate how the different estimation methods fare with such a design.

The data set contains *N* = 3,344 respondents and 74 items in total. The routing block contains two parallel forms with 17 items in each form. The three blocks in stage II contain 14, 13, and 13 items, respectively. Each person responded to 31 or 30 items out of 74. The items cover 5 different content domains, i.e., number properties and operations, measurement, geometry, data analysis statistics and probability, and algebra. The break down of items from each content domain in each form is presented in Table 8 by Wang et al. (2020). The content coverage is pretty balanced, which suggests a five-dimensional model to be appropriate. Hence, five-dimensional exploratory M2PL, M3PL, and M4PL models were fitted to the MST data using IWVAE, MHRM, and MCEM. We used the same algorithm, architecture, hyper-parameters, and stop criteria on IWVAE as in the simulation study except that we use a larger learning rate of 0.1 for ϕ, **A**, ***b*** and 0.01 for ***c***, ***d***.

First, we studied the estimates of the covariance matrix Σ_*x*_ and the comparison between the three methods. Due to the identifiability issue, in all models we assumed that covariance matrix of latent factors is Σ = **I** and conducted the promax rotation to estimate the correlation matrix $\hat{\text{R}}$. After rotation, we adjusted the sign of the correlation depending on the sign of *post-hoc* transformed ${\hat{\text{A}}}^{rf}$ as in the previous section. In particular, we flipped the sign of each column in ${\hat{\text{A}}}^{r}$ if its sum was negative, and did the same to the corresponding columns and rows in the $\hat{\text{R}}$. Table 9 shows estimated matrices under M4PL, M3PL, and M2PL, respectively. Under all settings, the correlation matrix recovered by IWVAE was similar to those from MCEM and MHRM, and a bit even closer to MHRM than MCEM did on M3PL and M2PL models.

**Table 9:** Comparison of estimated *R*_*θ*_ from different models on MST dataset.

**Model**	**IWVAE**	**MCEM**	**MHRM**
M4PL	$\left[\begin{array}{ccccc} 1.\\ 0.624 & 1.\\ 0.571 & 0.51 & 1.\\ 0.695 & 0.625 & 0.551 & 1.\\ 0.53 & 0.521 & 0.457 & 0.546 & 1. \end{array}\right]$	$\left[\begin{array}{ccccc} 1.\\ 0.628 & 1.\\ 0.616 & 0.604 & 1.\\ 0.595 & 0.599 & 0.543 & 1.\\ 0.655 & 0.655 & 0.621 & 0.626 & 1. \end{array}\right]$	/
M3PL	$\left[\begin{array}{ccccc} 1.\\ 0.585 & 1.\\ 0.557 & 0.699 & 1.\\ 0.531 & 0.671 & 0.653 & 1.\\ 0.47 & 0.52 & 0.521 & 0.496 & 1. \end{array}\right]$	$\left[\begin{array}{ccccc} 1.\\ 0.465 & 1.\\ 0.669 & 0.363 & 1.\\ 0.712 & 0.449 & 0.662 & 1.\\ 0.668 & 0.43 & 0.603 & 0.665 & 1. \end{array}\right]$	$\left[\begin{array}{ccccc} 1.\\ 0.581 & 1.\\ 0.52 & 0.589 & 1.\\ 0.565 & 0.689 & 0.582 & 1.\\ 0.48 & 0.586 & 0.494 & 0.61 & 1. \end{array}\right]$
M2PL	$\left[\begin{array}{ccccc} 1.\\ 0.74 & 1.\\ 0.622 & 0.628 & 1.\\ 0.58 & 0.529 & 0.515 & 1.\\ 0.594 & 0.593 & 0.507 & 0.451 & 1. \end{array}\right]$	$\left[\begin{array}{ccccc} 1.\\ 0.264 & 1.\\ 0.422 & 0.469 & 1.\\ 0.479 & 0.564 & 0.709 & 1.\\ 0.435 & 0.479 & 0.625 & 0.69 & 1. \end{array}\right]$	$\left[\begin{array}{ccccc} 1.\\ 0.518 & 1.\\ 0.593 & 0.584 & 1.\\ 0.548 & 0.548 & 0.618 & 1.\\ 0.551 & 0.601 & 0.624 & 0.638 & 1. \end{array}\right]$

Next, given that the true parameters are unknown, we evaluated the predictive performances of the three methods using a held-out validation. That is, we randomly marked 20% of items as *missing*, which played the role of held-out data, and used the remaining data to estimate our person and item parameters. That is, we used the estimated parameters to produce model-based predicted responses, compared them with the observed responses, and computed their consistency as a measure of accuracy. We computed such accuracy on both training data and held-out data. Higher accuracy indicates more alignment between model prediction and observed data. Meanwhile, we also used the estimated model parameters to compute log-likelihood, with a higher likelihood implying the estimated parameters may be better reflective of unknown truth. We reported accuracy and log-likelihood predicted by different methods on the training and held-out data in Table 10. To eliminate potential randomness in generating observed responses, 5 replications were done for each model, and we generated a different train and held-out data in each replication.

**Table 10:** Mean and SE of train and held-out accuracy/log-likelihood on MST dataset (over 5 replications).

**Method**	**Model**	**Train accuracy**	**Held-out accuracy**	**Train log-likelihood**	**Held-out log-likelihood**
IWVAE	M4PL	0.707 ± 0.001	0.704 ± 0.002	−0.531 ± 0.001	−0.539 ± 0.001
	M3PL	0.707 ± 0.000	0.706 ± 0.002	−0.530 ± 0.000	−0.537 ± 0.000
	M2PL	0.706 ± 0.001	0.703 ± 0.001	−0.531 ± 0.001	−0.539 ± 0.001
MCEM	M4PL	0.764 ± 0.001	0.693 ± 0.002	−0.481 ± 0.001	−0.603 ± 0.001
	M3PL	0.761 ± 0.000	0.697 ± 0.001	−0.482 ± 0.000	−0.599 ± 0.000
	M2PL	0.759 ± 0.001	0.697 ± 0.001	−0.485 ± 0.001	−0.589 ± 0.001
MHRM	M4PL	/	/	/	/
	M3PL	0.612 ± 0.003	0.613 ± 0.002	−0.682 ± 0.003	−0.683 ± 0.003
	M2PL	0.622 ± 0.003	0.623 ± 0.002	−0.662 ± 0.003	−0.664 ± 0.003

**Figure 3:**
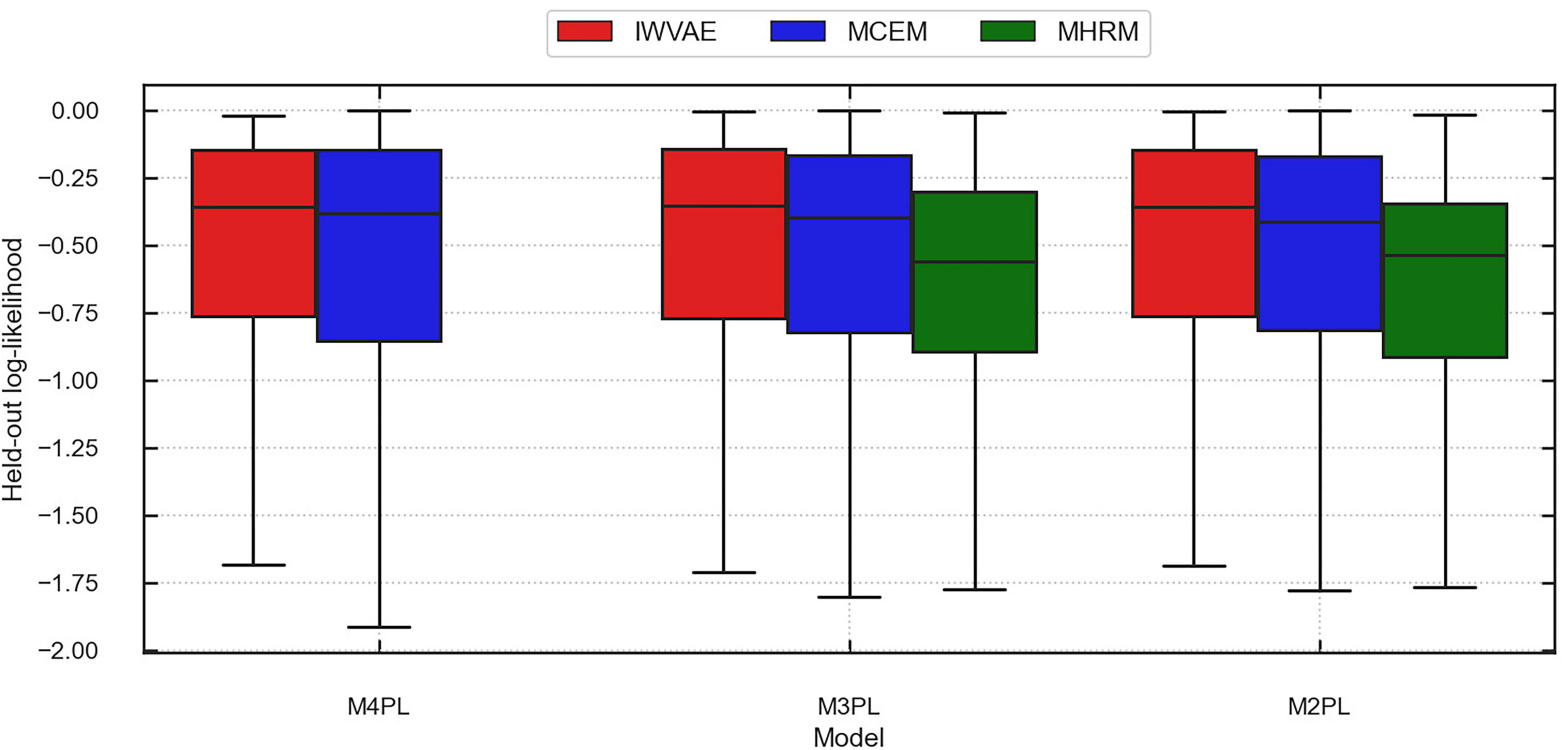
Predicted log-likelihood on held-out items using different methods (IWVAE, MHRM, MCEM) to fit different MIRT models on MST data from a randomly selected trial. Outlier predictions are removed.

**Figure 4:**
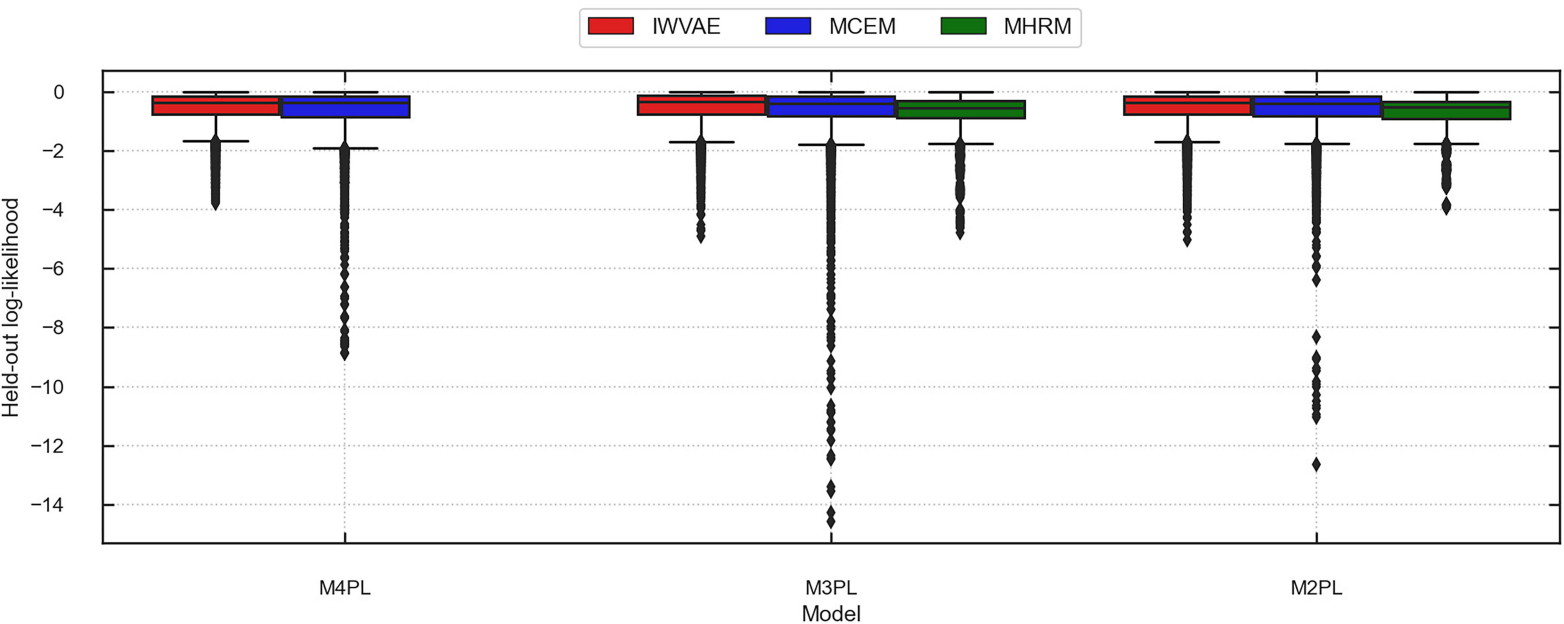
Predicted log-likelihood on held-out items using different methods (IWVAE, MHRM, MCEM) to fit different MIRT models on MST data from a randomly selected trial. Outlier predictions are kept.

Table 10 summarizes the averaged accuracy and log-likelihood (of each item) on the train and held-out sets, where values in parentheses are stand errors across 5 replications. In this experiment, IWVAE achieved the highest held-out accuracy and log-likelihood. Figures 3, 4 further showed the corresponding log-likelihood values of each item. First, we observed that IWVAE had much fewer outliers than MCEM; after removing outliers, IWVAE achieved the highest log-likelihood on three MIRT models. Moreover, among the three models, the held-out accuracy, training data log-likelihood, and held-out log-likelihood from IWVAE were the best for M3PL. This is expected in that the operational model for NAEP analysis is indeed 3PL.

## 5. Discussions

In this article, we extend a variational autoencoder estimation method (Urban and Bauer, 2021) for the parameter estimation of the M3PL and M4PL models. By approximating the intractable log-likelihood with variational techniques, it provides a computationally efficient and scalable method for the estimation of large-scale assessment data. Simulation studies demonstrate that the proposed method outperforms the widely used MHRM and MCEM methods in terms of parameter recovery and computation time in both M3PL and M4PL. The proposed method is also more robust with many fewer issues of convergence. That said, we do want to caution readers that a robust algorithm cannot compensate for a lack of data. For M3PL and M4PL to be estimated well, there needs to be enough data at the two extreme ends of the latent trait scales to help estimate the lower and upper asymptote adequately.

Although this study focuses on the exploratory item factor analysis, the proposed algorithm can be easily applied to the confirmatory item factor analysis, where certain entries of the loading matrix are set to be 0 by users. Such structural restrictions can be naturally incorporated into the estimation. In addition, it would be also of interest to further estimate the sparsity loading structure from the responses. This can be achieved by adding a lasso-type regularization term into the loss function (the marginal log-likelihood function), which would induce sparse estimation results from the regularized algorithms.

Finally, a few interesting problems are left for future investigations. Very recent works suggested that some aspects of our training strategy can be improved; for instance, Collier et al. (2021) revealed that the missing data can be handled better than zero-imputation; and Wang et al. (2021) indicated a possible direction of understanding and solving the posterior collapse, which was solved by a KL annealing stage in our proposed method. Moreover, this work does not directly study the estimation uncertainty of the VAE estimation procedure. It is interesting to further develop valid statistical procedures to make inferences for the corresponding estimation results. Such an important problem, however, still remains unaddressed for VAE and related deep learning methods in the machine learning and statistics literature.

## Data availability statement

The raw data supporting the conclusions of this article will be made available by the authors, without undue reservation.

## Author contributions

TL contributed to implementing the studies and writing the initial draft. GX contributed to mentoring, conceptualizing ideas, acquiring funding, and manuscript revision. CW contributed to conceptualizing ideas, acquiring funding, and manuscript revision. All authors contributed to the article and approved the submitted version.

## Funding

This study was partially supported by IES grant R305D200015, NSF grants SES-1846747 and NSF SES-2150601.

## Conflict of interest

The authors declare that the research was conducted in the absence of any commercial or financial relationships that could be construed as a potential conflict of interest.

## Publisher's note

All claims expressed in this article are solely those of the authors and do not necessarily represent those of their affiliated organizations, or those of the publisher, the editors and the reviewers. Any product that may be evaluated in this article, or claim that may be made by its manufacturer, is not guaranteed or endorsed by the publisher.

## Footnotes

^1^Here is a bit of abuse of notation: we use **1** to denote both the indicator function **1**(·) and its output.

^2^HPCC cannot be used due to the license issue of FlexMIRT.

^3^Unlike the mirt package, FlexMIRT only provides convergent estimates.

## References

[B1] Barton M. A. Lord F. M. (1981). An upper asymptote for the three-parameter logistic item-response model. ETS Res. Rep. Series 1981, i-8. 10.1002/j.2333-8504.1981.tb01255.x34002857

[B2] Bishop C. M. (2006). Pattern Recognition and Machine Learning (Information Science and Statistics). Berlin; Heidelberg: Springer-Verlag.

[B3] Blei D. M. Kucukelbir A. McAuliffe J. D. (2017). Variational Inference: a Review for Statisticians. J. Am. Stat. Assoc. 112, 859–877. 10.1080/01621459.2017.1285773

[B4] Bock R. D. Aitkin M. (1981). Marginal maximum likelihood estimation of item parameters: application of an EM algorithm. Psychometrika 46, 443–459. 10.1007/BF02293801

[B5] Burda Y. Grosse R. B. Salakhutdinov R. (2016). Importance weighted autoencoders, in ICLR (San Juan).

[B6] Cai L. (2010a). High-dimensional exploratory item factor analysis by a metropolis “hastings robbins” monro algorithm. Psychometrika 75, 33–57. 10.1007/s11336-009-9136-x33528784

[B7] Cai L. (2010b). Metropolis-hastings robbins-monro algorithm for confirmatory item factor analysis. J. Educ. Behav. Stat.35, 307–335. 10.3102/1076998609353115

[B8] Chalmers R. P. (2012). mirt: a multidimensional item response theory package for the R environment. J. Stat. Softw. 48, 1–29. 10.18637/jss.v048.i06

[B9] Chen Y. Li X. Zhang S. (2019). Joint maximum likelihood estimation for high-dimensional exploratory item response analysis. Psychometrika 84, 124–146. 10.1007/s11336-018-9646-5 30456747

[B10] Cho A. E. Wang C. Zhang X. Xu G. (2021). Gaussian variational estimation for multidimensional item response theory. Br. J. Math. Stat. Psychol. 74, 52–85. 10.1111/bmsp.12219 33064318

[B11] Cho A. E. Xiao J. Wang C. Xu G. (2022). Regularized variational estimation for exploratory item factor analysis. Psychometrika. 10.1007/s11336-022-09874-6 35831697

[B12] Chung S. Houts C. (2020). flexMIRT: a flexible modeling package for multidimensional item response models. Measurement Interdisc. Res. Perspect. 18, 40–54. 10.1080/15366367.2019.1693825

[B13] Collier M. Nazabal A. Williams C. K. I. (2021). VAEs in the presence of missing data. arXiv preprint arXiv:2006.05301.

[B14] Curi M. Converse G. A. Hajewski J. Oliveira S. (2019). Interpretable variational autoencoders for cognitive models, in 2019 International Joint Conference on Neural Networks (IJCNN) (Budapest: IEEE), 1–8.

[B15] Cybenko G. (1989). Approximation by superpositions of a sigmoidal function. Math. Control Signals Syst. 2, 303–314. 10.1007/BF02551274

[B16] Dempster A. P. Laird N. M. Rubin D. B. (1977). Maximum likelihood from incomplete data via the EM algorithm. J. R. Stat. Soc. B 39, 1–38.

[B17] Goodfellow I. Bengio Y. Courville A. (2016). Deep Learning. The MIT Press.

[B18] Gulrajani I. Kumar K. Ahmed F. Taiga A. A. Visin F. Vazquez D.. (2016). PixelVAE: a latent variable model for natural images. arXiv:1611.05013 [cs.LG]. 10.48550/arXiv.1611.05013

[B19] Hambleton R. K. Swaminathan H. (1985). Item Response Theory: Principles and Applications. Boston, MA: Kluwer Academic.

[B20] Hendrickson A. E. White P. O. (1964). Promax: a quick method for rotation to oblique simple structure. Br. J. Stat. Psychol. 17, 65–70. 10.1111/j.2044-8317.1964.tb00244.x

[B21] Hornik K. (1991). Approximation capabilities of multilayer feedforward networks. Neural Networks 4, 251–257. 10.1016/0893-6080(91)90009-T

[B22] Hui F. K. Warton D. I. Ormerod J. T. Haapaniemi V. Taskinen S. (2017). Variational approximations for generalized linear latent variable models. J. Comput. Graph. Stat. 26, 35–43. 10.1080/10618600.2016.116470831042745

[B23] Jeon M. Rijmen F. Rabe-Hesketh S. (2017). A variational maximization-maximization algorithm for generalized linear mixed models with crossed random effects. Psychometrika 82, 693–716. 10.1007/s11336-017-9555-z 28247165

[B24] Kingma D. P. Salimans T. Jozefowicz R. Chen X. Sutskever I. Welling M. (2016). Improving variational inference with inverse autoregressive flow, Neural Information Processing Systems (Barcelona), 29.

[B25] Kingma D. P. Welling M. (2014). Auto-encoding variational bayes. arXiv preprint arXiv:1312.6114. 10.48550/arXiv.1312.6114

[B26] Kingma D. P. Welling M. (2019). An introduction to variational autoencoders. Foundat. Trends Mach. Learn. 12, 307–392. 10.1561/2200000056

[B27] Kucukelbir A. Tran D. Ranganath R. Gelman A. Blei D. M. (2017). Automatic differentiation variational inference. J. Mach. Learn. Res. 18, 1–45. 10.48550/arXiv.1603.00788

[B28] Lindstrom M. J. Bates D. M. (1988). Newton-raphson and EM algorithms for linear mixed-effects models for repeated-measures data. J. Am. Stat. Assoc. 83, 1014–1022.

[B29] Loken E. Rulison K. L. (2010). Estimation of a four-parameter item response theory model. Br. J. Math. Stat. Psychol. 63, 509–525. 10.1348/000711009X474502 20030965

[B30] McCulloch C. (1997). Maximum likelihood algorithms for generalized linear mixed models. J. Am. Stat. Assoc. 92, 162–170. 10.1080/01621459.1997.10473613

[B31] McDonald R. P. (1967). Nonlinear factor analysis. Psychometric. Monogr. 15, 167–167.

[B32] Meng X. Xu G. Zhang J. Tao J. (2020). Marginalized maximum a posteriori estimation for the four-parameter logistic model under a mixture modelling framework. Br. J. Math. Stat. Psychol. 73, 51–82. 10.1111/bmsp.12185 31552688

[B33] Nair V. Hinton G. E. (2010). Rectified linear units improve restricted boltzmann machines, in International Conference on Machine Learning (Haifa).

[B34] Natesan P. Nandakumar R. Minka T. Rubright J. D. (2016). Bayesian prior choice in IRT estimation using MCMC and variational bayes. Front. Psychol. 7, 1422. 10.3389/fpsyg.2016.01422 27729878 PMC5037236

[B35] Nazabal A. Olmos P. M. Ghahramani Z. Valera I. (2020). Handling incomplete heterogeneous data using VAEs. Pattern Recognit. 107, 107501. 10.1016/j.patcog.2020.107501

[B36] Ogasawara H. (2002). Stable response functions with unstable item parameter estimates. Appl. Psychol. Meas. 26, 239–254. 10.1177/0146621602026003001

[B37] Papamakarios G. Nalisnick E. Rezende D. J. Mohamed S. Lakshminarayanan B. (2021). Normalizing flows for probabilistic modeling and inference. J. Mach. Learn. Res. 22, 1–64. 10.48550/arXiv.1912.0276232200210

[B38] Pascanu R. Mikolov T. Bengio Y. (2013). On the difficulty of training recurrent neural networks, in International Conference on Machine Learning (PMLR), 1310–1318.

[B39] Paszke A. Gross S. Massa F. Lerer A. Bradbury J. Chanan G.. (2019). Pytorch: an imperative style, high-performance deep learning library, in Neural Information Processing Systems Vol. 32 (Vancouver, CA).

[B40] Rainforth T. Kosiorek A. Le T. A. Maddison C. Igl M. Wood F.. (2018). Tighter variational bounds are not necessarily better, in International Conference on Machine Learning (Stockholm), 4277–4285.

[B41] Reckase M. D. (2009). Multidimensional item response theory models, in Multidimensional Item Response Theory (Springer), 79–112.

[B42] Reise S. P. Waller N. G. (2003). How many IRT parameters does it take to model psychopathology items? Psychol. Methods 8, 164. 10.1037/1082-989x.8.2.164 12924813

[B43] Rijmen F. Jeon M. (2013). Fitting an item response theory model with random item effects across groups by a variational approximation method. Ann. Operat. Res. 206, 647–662. 10.1007/s10479-012-1181-7

[B44] Sønderby C. K. Raiko T. Maaløe L. Sønderby S. K. Winther O. (2016). Ladder variational autoencoders, in Neural Information Processing Systems, Vol. 29 (Barcelona).

[B45] Sonoda S. Murata N. (2017). Neural network with unbounded activation functions is universal approximator. Appl. Comput. Harmon Anal. 43, 233–268. 10.1016/j.acha.2015.12.005

[B46] Tierney L. Kadane J. B. (1986). Accurate approximations for posterior moments and marginal densities. J. Am. Stat. Assoc. 81, 82–86. 10.1080/01621459.1986.10478240

[B47] Tucker G. Lawson D. Gu S. Maddison C. J. (2018). Doubly reparameterized gradient estimators for monte carlo objectives, in International Conference on Learning Representations (Vancouver, CA).

[B48] Urban C. J. Bauer D. J. (2021). A deep learning algorithm for high-dimensional exploratory item factor analysis. Psychometrika 86, 1–29. 10.1007/s11336-021-09748-3 33528784

[B49] von Davier M. Sinharay S. (2010). Stochastic approximation methods for latent regression item response models. J. Educ. Behav. Stat. 35, 174–193. 10.3102/1076998609346970

[B50] Waller N. G. Feuerstahler L. (2017). Bayesian modal estimation of the four-parameter item response model in real, realistic, and idealized data sets. Multivariate Behav Res. 52, 350–370. 10.1080/00273171.2017.1292893 28306347

[B51] Waller N. G. Reise S. (2010). Measuring psychopathology with non-standard IRT models: fitting the four-parameter model to the MMPI, in Measuring Psychological Constructs With Model-Based Approaches (American Psychological Association), 147–173.

[B52] Wang C. Chen P. Jiang S. (2020). Item calibration methods with multiple subscale multistage testing. J. Educ. Meas. 57, 3–28. 10.1111/jedm.12241

[B53] Wang Y. Blei D. Cunningham J. P. (2021). Posterior collapse and latent variable non-identifiability, in Neural Information Processing Systems, Vol. 34.

[B54] Wolfinger R. O'connell M. (1993). Generalized linear mixed models a pseudo-likelihood approach. J. Stat. Comput. Simul. 48, 233–243.

[B55] Wu M. Davis R. L. Domingue B. W. Piech C. Goodman N. (2020). Variational item response theory: fast, accurate, and expressive. arXiv preprint arXiv:2002.00276. 10.48550/arXiv.2002.00276

[B56] Yen Y.-C. Ho R.-G. Laio W.-W. Chen L.-J. Kuo C.-C. (2012). An empirical evaluation of the slip correction in the four parameter logistic models with computerized adaptive testing. Appl. Psychol. Meas. 36, 75–87. 10.1177/0146621611432862

[B57] Zhang A. Lipton Z. C. Li M. Smola A. J. (2021). Dive into deep learning. arXiv preprint arXiv:2106.11342.

[B58] Zhang S. Chen Y. Liu Y. (2020). An improved stochastic EM Algorithm for large-scale full-information item factor analysis. Br. J. Math. Stat. Psychol. 73, 44–71. 10.1111/bmsp.12153 30511445

