# Supplementary material for: Estimating three- and four-parameter MIRT models with importance-weighted sampling enhanced variational auto-encoder
Source: Front Psychol. 2022 Aug 15;13:935419. doi: 10.3389/fpsyg.2022.935419 (PMC9421264; doi:10.3389/fpsyg.2022.935419)
Supplement: Supplementary file 1 [file Presentation_1.pdf]

Supplemental Materials

1 Q-matrix sparsity estimation

In this section we evaluate the sparsity structure estimation of Q-matrix $\mathbf{A}$ by MCEM, MHRM, and IWVAE. Towards this goal, we follow Cho et al. (accepted) and apply the CF-Quartimax rotation to estimated Q-matrix $\hat{\mathbf{A}}^{rf}$ constructed in section 3.1.

To be specific, let $\hat{\mathbf{\Sigma}}^{rf}$ be the estimated population covariance matrix corresponding to $\hat{\mathbf{A}}^{rf}$, we first transformed $\hat{\boldsymbol{a}}_j^{rf} \in \mathbb{R}^{K\times 1}$ to a standardized factor loading by

$$\boldsymbol{\psi}_j = \mathbf{U}^{-1}\hat{\boldsymbol{a}}_j^{rf}, \quad \text{where} \quad \mathbf{U}^\top\mathbf{U} = \left(\mathbf{I} + \left((\hat{\boldsymbol{a}}^{rf})_j^\top \hat{\boldsymbol{a}}_j^{rf}\right)\hat{\mathbf{\Sigma}}^{rf}\right).$$

Next, if $|\psi_{jk}| > 0.3$, we assume that the j-th item is loaded on the k-th factor (Henson and Roberts, 2006; Costello and Osborne, 2005).

We reported averages of standard errors of accuracy, sensitivity, specificity, and F1 scores of MCEM, MHRM, and IWVAE on synthetic datasets in Tables 1 to 8. Compared with MCEM, IWVAE had higher accuracy and comparable F1 score, and nearly on all experiments, IWVAE had higher specificity and lower sensitivity. These together indicate that IWAE was more conservative than MCEM and produced more sparse estimation. Compared with MHRM, as only convergent results were reported (by FlexMIRT), it again performed better than MCEM and IWVAE.

N, J	Item Structure	Model	Accuracy	Sensitivity	Specificity	F1	Success Rates
500 100	Between	MCEM	0.542 ± 0.003	**0.571 ± 0.005**	0.535 ± 0.003	**0.333 ± 0.003**	1.00
		MHRM	/	/	/	/	/
		IWVAE	**0.593 ± 0.002**	0.491 ± 0.005	**0.618 ± 0.002**	0.326 ± 0.003	1.00
	Within	MCEM	0.548 ± 0.003	**0.557 ± 0.004**	0.542 ± 0.003	**0.496 ± 0.003**	1.00
		MHRM	/	/	/	/	/
		IWVAE	**0.575 ± 0.002**	0.488 ± 0.003	**0.632 ± 0.002**	0.479 ± 0.003	1.00
1000 100	Between	MCEM	0.542 ± 0.002	**0.602 ± 0.005**	0.527 ± 0.002	**0.344 ± 0.003**	1.00
		MHRM	/	/	/	/	/
		IWVAE	**0.652 ± 0.002**	0.457 ± 0.005	**0.701 ± 0.002**	**0.344 ± 0.004**	1.00
	Within	MCEM	0.560 ± 0.003	**0.573 ± 0.004**	0.551 ± 0.003	**0.510 ± 0.003**	1.00
		MHRM	/	/	/	/	/
		IWVAE	**0.610 ± 0.003**	0.480 ± 0.004	**0.697 ± 0.003**	0.496 ± 0.004	1.00
5000 100	Between	MCEM	0.614 ± 0.003	**0.622 ± 0.006**	0.612 ± 0.004	**0.392 ± 0.004**	1.00
		MHRM	/	/	/	/	/
		IWVAE	**0.759 ± 0.003**	0.388 ± 0.006	**0.852 ± 0.003**	0.392 ± 0.006	1.00
	Within	MCEM	0.584 ± 0.003	**0.538 ± 0.004**	0.615 ± 0.003	**0.509 ± 0.004**	1.00
		MHRM	/	/	/	/	/
		IWVAE	**0.642 ± 0.004**	0.406 ± 0.004	**0.800 ± 0.004**	0.476 ± 0.005	1.00
10000 100	Between	MCEM	0.609 ± 0.005	**0.578 ± 0.008**	0.616 ± 0.006	0.373 ± 0.006	1.00
		MHRM	/	/	/	/	/
		IWVAE	**0.758 ± 0.003**	0.400 ± 0.007	**0.848 ± 0.002**	**0.398 ± 0.006**	1.00
	Within	MCEM	0.577 ± 0.004	**0.519 ± 0.004**	0.616 ± 0.005	0.496 ± 0.004	1.00
		MHRM	/	/	/	/	/
		IWVAE	**0.660 ± 0.004**	0.428 ± 0.004	**0.815 ± 0.004**	**0.502 ± 0.005**	1.00

Table 1: Mean and standard error of **A** sparsity estimate on M4PL models under **single** regime setting. Factors are diagonal. **Between** item structure: each item depends on 1 factor. **Within** item structure: each item depends on 2 factors

References

A. E. Cho, J. Xiao, C. Wang, and G. Xu. Regularized Variational Estimation for Exploratory Item Factor Analysis. *Psychometrika*, accepted.

A. B. Costello and J. Osborne. Best practices in exploratory factor analysis: Four recommendations for getting the most from your analysis. *Practical assessment, research, and evaluation*, 10(1):7, 2005.

R. K. Henson and J. K. Roberts. Use of Exploratory Factor Analysis in Published Research: Common Errors and Some Comment on Improved Practice. *Educational and Psychological measurement*, 66(3):393–416, 2006.

N, J	Item Structure	Model	Accuracy	Sensitivity	Specificity	F1	Success Rates
500 100	Between	MCEM	0.542 ± 0.003	**0.571 ± 0.005**	0.535 ± 0.003	**0.333 ± 0.003**	1.00
		MHRM	/	/	/	/	/
		IWVAE	**0.593 ± 0.002**	0.491 ± 0.005	**0.618 ± 0.002**	0.326 ± 0.003	1.00
	Within	MCEM	0.548 ± 0.003	**0.557 ± 0.004**	0.542 ± 0.003	**0.496 ± 0.003**	1.00
		MHRM	/	/	/	/	/
		IWVAE	**0.575 ± 0.002**	0.488 ± 0.003	**0.632 ± 0.002**	0.479 ± 0.003	1.00
1000 200	Between	MCEM	0.546 ± 0.003	**0.544 ± 0.005**	0.546 ± 0.004	**0.324 ± 0.002**	1.00
		MHRM	/	/	/	/	/
		IWVAE	**0.640 ± 0.002**	0.404 ± 0.005	**0.699 ± 0.002**	0.310 ± 0.004	1.00
	Within	MCEM	0.558 ± 0.002	**0.577 ± 0.003**	0.545 ± 0.002	**0.511 ± 0.002**	1.00
		MHRM	/	/	/	/	/
		IWVAE	**0.610 ± 0.003**	0.475 ± 0.003	**0.700 ± 0.003**	0.494 ± 0.004	1.00
5000 300	Between	MCEM	0.631 ± 0.004	**0.524 ± 0.007**	0.658 ± 0.004	0.363 ± 0.005	1.00
		MHRM	/	/	/	/	/
		IWVAE	**0.724 ± 0.003**	0.451 ± 0.006	**0.793 ± 0.002**	**0.396 ± 0.006**	1.00
	Within	MCEM	0.605 ± 0.003	**0.515 ± 0.004**	0.665 ± 0.003	**0.510 ± 0.004**	1.00
		MHRM	/	/	/	/	/
		IWVAE	**0.637 ± 0.003**	0.439 ± 0.004	**0.769 ± 0.003**	0.491 ± 0.005	1.00
10000 500	Between	MCEM	0.640 ± 0.006	**0.506 ± 0.006**	0.674 ± 0.007	0.363 ± 0.005	1.00
		MHRM	/	/	/	/	/
		IWVAE	**0.724 ± 0.003**	0.418 ± 0.007	**0.800 ± 0.002**	**0.377 ± 0.006**	1.00
	Within	MCEM	0.595 ± 0.004	**0.528 ± 0.004**	0.640 ± 0.005	**0.511 ± 0.004**	1.00
		MHRM	/	/	/	/	/
		IWVAE	**0.623 ± 0.003**	0.411 ± 0.004	**0.765 ± 0.003**	0.466 ± 0.005	1.00

Table 2: Mean and standard error of **A** sparsity estimate on M4PL models under **double** regime setting. Factors are diagonal. **Between** item structure: each item depends on 1 factor. **Within** item structure: each item depends on 2 factors

N, J	Item Structure	Model	Accuracy	Sensitivity	Specificity	F1	Success Rates
500 100	Between	MCEM	0.539 ± 0.002	0.533 ± 0.005	0.540 ± 0.002	0.316 ± 0.003	1.00
		MHRM	**0.934 ± 0.032**	**0.701 ± 0.165**	**0.993 ± 0.002**	**0.698 ± 0.162**	0.35
		IWVAE	0.604 ± 0.002	0.486 ± 0.005	0.633 ± 0.002	0.329 ± 0.004	1.00
	Within	MCEM	0.551 ± 0.003	**0.526 ± 0.004**	0.567 ± 0.003	0.483 ± 0.003	1.00
		MHRM	**0.749 ± 0.020**	0.523 ± 0.069	**0.900 ± 0.014**	**0.592 ± 0.078**	0.40
		IWVAE	0.594 ± 0.003	0.516 ± 0.004	0.646 ± 0.003	0.504 ± 0.003	1.00
1000 100	Between	MCEM	0.569 ± 0.002	0.548 ± 0.006	0.574 ± 0.002	0.337 ± 0.004	1.00
		MHRM	**0.965 ± 0.030**	**0.835 ± 0.151**	**0.998 ± 0.000**	**0.832 ± 0.148**	0.30
		IWVAE	0.656 ± 0.002	0.472 ± 0.006	0.703 ± 0.003	0.354 ± 0.004	1.00
	Within	MCEM	0.568 ± 0.003	**0.513 ± 0.004**	0.605 ± 0.003	0.487 ± 0.004	1.00
		MHRM	**0.753 ± 0.022**	0.463 ± 0.067	**0.946 ± 0.008**	**0.548 ± 0.077**	0.65
		IWVAE	0.633 ± 0.004	0.498 ± 0.005	0.722 ± 0.004	0.520 ± 0.005	1.00
5000 100	Between	MCEM	0.651 ± 0.004	0.567 ± 0.008	0.673 ± 0.003	0.394 ± 0.006	1.00
		MHRM	**0.932 ± 0.039**	**0.672 ± 0.190**	**0.998 ± 0.002**	**0.676 ± 0.187**	0.30
		IWVAE	0.753 ± 0.003	0.468 ± 0.007	0.824 ± 0.003	0.431 ± 0.006	1.00
	Within	MCEM	0.603 ± 0.004	0.487 ± 0.005	0.680 ± 0.003	0.495 ± 0.005	1.00
		MHRM	**0.796 ± 0.003**	**0.584 ± 0.003**	**0.937 ± 0.005**	**0.696 ± 0.004**	0.90
		IWVAE	0.645 ± 0.004	0.459 ± 0.005	0.768 ± 0.004	0.508 ± 0.006	1.00
10000 100	Between	MCEM	0.682 ± 0.003	0.539 ± 0.007	0.718 ± 0.003	0.405 ± 0.006	1.00
		MHRM	**0.984 ± 0.014**	**0.930 ± 0.067**	**0.998 ± 0.001**	**0.928 ± 0.066**	0.70
		IWVAE	0.753 ± 0.003	0.432 ± 0.007	0.834 ± 0.003	0.412 ± 0.006	1.00
	Within	MCEM	0.612 ± 0.004	0.502 ± 0.005	0.686 ± 0.003	0.509 ± 0.004	1.00
		MHRM	**0.768 ± 0.012**	**0.554 ± 0.036**	**0.911 ± 0.005**	**0.641 ± 0.041**	0.75
		IWVAE	0.661 ± 0.005	0.471 ± 0.005	0.787 ± 0.005	0.526 ± 0.006	1.00

Table 3: Mean and standard error of **A** sparsity estimate on M3PL models under **single** regime setting. Factors are diagonal. **Between** item structure: each item depends on 1 factor. **Within** item structure: each item depends on 2 factors

N, J	Item Structure	Model	Accuracy	Sensitivity	Specificity	F1	Success Rates
500 100	Between	MCEM	0.539 ± 0.002	0.533 ± 0.005	0.540 ± 0.002	0.316 ± 0.003	1.00
		MHRM	**0.934 ± 0.032**	**0.701 ± 0.165**	**0.993 ± 0.002**	**0.698 ± 0.162**	0.35
		IWVAE	0.604 ± 0.002	0.486 ± 0.005	0.633 ± 0.002	0.329 ± 0.004	1.00
	Within	MCEM	0.551 ± 0.003	**0.526 ± 0.004**	0.567 ± 0.003	0.483 ± 0.003	1.00
		MHRM	**0.749 ± 0.020**	0.523 ± 0.069	**0.900 ± 0.014**	**0.592 ± 0.078**	0.40
		IWVAE	0.594 ± 0.003	0.516 ± 0.004	0.646 ± 0.003	0.504 ± 0.003	1.00
1000 200	Between	MCEM	0.560 ± 0.002	**0.530 ± 0.005**	0.568 ± 0.002	0.325 ± 0.003	1.00
		MHRM	/	/	/	/	0.00
		IWVAE	**0.648 ± 0.002**	0.468 ± 0.005	**0.692 ± 0.002**	**0.347 ± 0.004**	1.00
	Within	MCEM	0.574 ± 0.003	**0.526 ± 0.004**	0.606 ± 0.003	0.497 ± 0.004	1.00
		MHRM	/	/	/	/	0.00
		IWVAE	**0.609 ± 0.003**	0.490 ± 0.004	**0.688 ± 0.003**	**0.501 ± 0.004**	1.00
5000 300	Between	MCEM	0.678 ± 0.003	**0.494 ± 0.009**	0.724 ± 0.003	0.380 ± 0.006	1.00
		MHRM	/	/	/	/	0.00
		IWVAE	**0.712 ± 0.003**	0.467 ± 0.008	**0.773 ± 0.002**	**0.394 ± 0.007**	1.00
	Within	MCEM	0.614 ± 0.004	**0.496 ± 0.005**	0.693 ± 0.004	**0.507 ± 0.005**	1.00
		MHRM	/	/	/	/	0.00
		IWVAE	**0.631 ± 0.004**	0.462 ± 0.005	**0.743 ± 0.004**	0.500 ± 0.005	1.00
10000 500	Between	MCEM	0.696 ± 0.003	**0.492 ± 0.008**	0.747 ± 0.003	**0.393 ± 0.006**	1.00
		MHRM	/	/	/	/	0.00
		IWVAE	**0.710 ± 0.003**	0.449 ± 0.007	**0.775 ± 0.003**	0.382 ± 0.006	1.00
	Within	MCEM	0.615 ± 0.004	**0.496 ± 0.005**	0.694 ± 0.004	**0.508 ± 0.005**	1.00
		MHRM	/	/	/	/	0.00
		IWVAE	**0.622 ± 0.004**	0.444 ± 0.006	**0.740 ± 0.004**	0.484 ± 0.006	1.00

Table 4: Mean and standard error of **A** sparsity estimate on M3PL models under **double** regime setting. Factors are diagonal. **Between** item structure: each item depends on 1 factor. **Within** item structure: each item depends on 2 factors

N, J	Item Structure	Model	Accuracy	Sensitivity	Specificity	F1	Success Rates
500 100	Between	MCEM	0.526 ± 0.002	**0.568 ± 0.005**	0.515 ± 0.003	0.324 ± 0.003	1.00
		MHRM	/	/	/	/	/
		IWVAE	**0.605 ± 0.002**	0.508 ± 0.005	**0.629 ± 0.002**	**0.340 ± 0.003**	1.00
	Within	MCEM	0.526 ± 0.003	**0.563 ± 0.004**	0.501 ± 0.003	**0.487 ± 0.003**	1.00
		MHRM	/	/	/	/	/
		IWVAE	**0.568 ± 0.002**	0.502 ± 0.003	**0.612 ± 0.002**	0.482 ± 0.003	1.00
1000 100	Between	MCEM	0.535 ± 0.002	**0.585 ± 0.005**	0.522 ± 0.002	0.335 ± 0.002	1.00
		MHRM	/	/	/	/	/
		IWVAE	**0.648 ± 0.002**	0.443 ± 0.005	**0.700 ± 0.003**	**0.335 ± 0.003**	1.00
	Within	MCEM	0.531 ± 0.003	**0.561 ± 0.004**	0.512 ± 0.003	0.489 ± 0.003	1.00
		MHRM	/	/	/	/	/
		IWVAE	**0.593 ± 0.003**	0.506 ± 0.004	**0.650 ± 0.003**	**0.498 ± 0.004**	1.00
5000 100	Between	MCEM	0.566 ± 0.003	**0.607 ± 0.005**	0.556 ± 0.002	**0.359 ± 0.003**	1.00
		MHRM	/	/	/	/	/
		IWVAE	**0.733 ± 0.003**	0.363 ± 0.007	**0.826 ± 0.002**	0.352 ± 0.006	1.00
	Within	MCEM	0.552 ± 0.003	**0.552 ± 0.004**	0.552 ± 0.003	**0.496 ± 0.003**	1.00
		MHRM	/	/	/	/	/
		IWVAE	**0.629 ± 0.003**	0.435 ± 0.005	**0.759 ± 0.004**	0.484 ± 0.004	1.00
10000 100	Between	MCEM	0.573 ± 0.003	**0.550 ± 0.006**	0.579 ± 0.004	0.341 ± 0.003	1.00
		MHRM	/	/	/	/	/
		IWVAE	**0.739 ± 0.002**	0.357 ± 0.006	**0.834 ± 0.002**	**0.353 ± 0.005**	1.00
	Within	MCEM	0.554 ± 0.003	**0.543 ± 0.005**	0.561 ± 0.004	0.493 ± 0.004	1.00
		MHRM	/	/	/	/	/
		IWVAE	**0.649 ± 0.003**	0.456 ± 0.005	**0.778 ± 0.004**	**0.509 ± 0.005**	1.00

Table 5: Mean and standard error of **A** sparsity estimate on M4PL models under **single** regime setting. Factors are correlated. **Between** item structure: each item depends on 1 factor. **Within** item structure: each item depends on 2 factors

N, J	Item Structure	Model	Accuracy	Sensitivity	Specificity	F1	Success Rates
500 100	Between	MCEM	0.526 ± 0.002	**0.568 ± 0.005**	0.515 ± 0.003	0.324 ± 0.003	1.00
		MHRM	/	/	/	/	/
		IWVAE	**0.605 ± 0.002**	0.508 ± 0.005	**0.629 ± 0.002**	**0.340 ± 0.003**	1.00
	Within	MCEM	0.526 ± 0.003	**0.563 ± 0.004**	0.501 ± 0.003	**0.487 ± 0.003**	1.00
		MHRM	/	/	/	/	/
		IWVAE	**0.568 ± 0.002**	0.502 ± 0.003	**0.612 ± 0.002**	0.482 ± 0.003	1.00
1000 200	Between	MCEM	0.517 ± 0.002	**0.560 ± 0.005**	0.507 ± 0.003	**0.317 ± 0.002**	1.00
		MHRM	/	/	/	/	/
		IWVAE	**0.625 ± 0.001**	0.422 ± 0.003	**0.676 ± 0.001**	0.310 ± 0.002	1.00
	Within	MCEM	0.535 ± 0.002	**0.574 ± 0.003**	0.509 ± 0.002	**0.497 ± 0.002**	1.00
		MHRM	/	/	/	/	/
		IWVAE	**0.587 ± 0.002**	0.488 ± 0.003	**0.653 ± 0.003**	0.486 ± 0.003	1.00
5000 300	Between	MCEM	0.588 ± 0.002	**0.567 ± 0.005**	0.594 ± 0.002	**0.355 ± 0.003**	1.00
		MHRM	/	/	/	/	/
		IWVAE	**0.700 ± 0.002**	0.406 ± 0.005	**0.773 ± 0.002**	0.351 ± 0.004	1.00
	Within	MCEM	0.572 ± 0.003	**0.546 ± 0.004**	0.589 ± 0.003	**0.505 ± 0.003**	1.00
		MHRM	/	/	/	/	/
		IWVAE	**0.611 ± 0.004**	0.469 ± 0.004	**0.706 ± 0.004**	0.491 ± 0.004	1.00
10000 500	Between	MCEM	0.605 ± 0.003	**0.504 ± 0.006**	0.630 ± 0.004	0.338 ± 0.004	1.00
		MHRM	/	/	/	/	/
		IWVAE	**0.706 ± 0.002**	0.395 ± 0.006	**0.783 ± 0.002**	**0.349 ± 0.005**	1.00
	Within	MCEM	0.553 ± 0.003	**0.520 ± 0.004**	0.575 ± 0.004	0.482 ± 0.003	1.00
		MHRM	/	/	/	/	/
		IWVAE	**0.628 ± 0.003**	0.449 ± 0.004	**0.747 ± 0.003**	**0.491 ± 0.005**	1.00

Table 6: Mean and standard error of **A** sparsity estimate on M4PL models under **double** regime setting. Factors are correlated. **Between** item structure: each item depends on 1 factor. **Within** item structure: each item depends on 2 factors

N, J	Item Structure	Model	Accuracy	Sensitivity	Specificity	F1	Success Rates
500 100	Between	MCEM	0.540 ± 0.002	0.538 ± 0.005	0.541 ± 0.002	0.319 ± 0.003	1.00
		MHRM	**0.810 ± 0.000**	**0.810 ± 0.000**	**0.810 ± 0.000**	**0.630 ± 0.000**	0.05
		IWVAE	0.597 ± 0.002	0.489 ± 0.005	0.624 ± 0.002	0.327 ± 0.003	1.00
	Within	MCEM	0.529 ± 0.003	0.529 ± 0.003	0.530 ± 0.003	0.474 ± 0.003	1.00
		MHRM	**0.720 ± 0.005**	**0.579 ± 0.006**	**0.815 ± 0.008**	**0.624 ± 0.005**	0.45
		IWVAE	0.569 ± 0.003	0.518 ± 0.003	0.603 ± 0.003	0.490 ± 0.003	1.00
1000 100	Between	MCEM	0.559 ± 0.002	**0.542 ± 0.004**	0.563 ± 0.002	0.330 ± 0.003	1.00
		MHRM	**0.821 ± 0.008**	0.478 ± 0.169	**0.906 ± 0.033**	**0.405 ± 0.137**	0.25
		IWVAE	0.652 ± 0.002	0.445 ± 0.005	0.703 ± 0.003	0.338 ± 0.003	1.00
	Within	MCEM	0.542 ± 0.003	0.533 ± 0.004	0.549 ± 0.003	0.482 ± 0.003	1.00
		MHRM	**0.751 ± 0.009**	**0.666 ± 0.012**	**0.807 ± 0.007**	**0.681 ± 0.012**	0.80
		IWVAE	0.608 ± 0.003	0.490 ± 0.004	0.686 ± 0.004	0.500 ± 0.004	1.00
5000 100	Between	MCEM	0.648 ± 0.003	0.560 ± 0.006	0.670 ± 0.002	0.389 ± 0.005	1.00
		MHRM	**0.886 ± 0.004**	**0.818 ± 0.009**	**0.903 ± 0.003**	**0.742 ± 0.008**	0.75
		IWVAE	0.722 ± 0.003	0.418 ± 0.007	0.797 ± 0.002	0.374 ± 0.006	1.00
	Within	MCEM	0.559 ± 0.003	0.529 ± 0.004	0.579 ± 0.003	0.489 ± 0.004	1.00
		MHRM	**0.671 ± 0.004**	**0.542 ± 0.033**	**0.758 ± 0.015**	**0.554 ± 0.031**	0.80
		IWVAE	0.627 ± 0.004	0.494 ± 0.006	0.715 ± 0.005	0.514 ± 0.005	1.00
10000 100	Between	MCEM	0.655 ± 0.003	0.539 ± 0.008	0.683 ± 0.003	0.385 ± 0.006	1.00
		MHRM	**0.947 ± 0.007**	**0.968 ± 0.014**	**0.942 ± 0.006**	**0.880 ± 0.015**	0.65
		IWVAE	0.732 ± 0.002	0.390 ± 0.006	0.817 ± 0.002	0.367 ± 0.005	1.00
	Within	MCEM	0.571 ± 0.003	0.533 ± 0.005	0.596 ± 0.003	0.498 ± 0.004	1.00
		MHRM	**0.681 ± 0.016**	**0.564 ± 0.022**	**0.759 ± 0.013**	**0.586 ± 0.022**	0.40
		IWVAE	0.630 ± 0.004	0.477 ± 0.006	0.732 ± 0.005	0.507 ± 0.006	1.00

Table 7: Mean and standard error of **A** sparsity estimate on M3PL models under **single** regime setting. Factors are correlated. **Between** item structure: each item depends on 1 factor. **Within** item structure: each item depends on 2 factors

N, J	Item Structure	Model	Accuracy	Sensitivity	Specificity	F1	Success Rates
500 100	Between	MCEM	0.540 ± 0.002	0.538 ± 0.005	0.541 ± 0.002	0.319 ± 0.003	1.00
		MHRM	**0.810 ± 0.000**	**0.810 ± 0.000**	**0.810 ± 0.000**	**0.630 ± 0.000**	0.05
		IWVAE	0.597 ± 0.002	0.489 ± 0.005	0.624 ± 0.002	0.327 ± 0.003	1.00
	Within	MCEM	0.529 ± 0.003	0.529 ± 0.003	0.530 ± 0.003	0.474 ± 0.003	1.00
		MHRM	**0.720 ± 0.005**	**0.579 ± 0.006**	**0.815 ± 0.008**	**0.624 ± 0.005**	0.45
		IWVAE	0.569 ± 0.003	0.518 ± 0.003	0.603 ± 0.003	0.490 ± 0.003	1.00
1000 200	Between	MCEM	0.555 ± 0.002	**0.513 ± 0.005**	0.565 ± 0.002	0.316 ± 0.003	1.00
		MHRM	/	/	/	/	0.00
		IWVAE	**0.633 ± 0.002**	0.461 ± 0.004	**0.675 ± 0.002**	**0.334 ± 0.003**	1.00
	Within	MCEM	0.552 ± 0.003	**0.537 ± 0.004**	0.562 ± 0.003	0.490 ± 0.003	1.00
		MHRM	/	/	/	/	0.00
		IWVAE	**0.579 ± 0.003**	0.511 ± 0.004	**0.625 ± 0.003**	**0.493 ± 0.004**	1.00
5000 300	Between	MCEM	0.650 ± 0.003	**0.505 ± 0.006**	0.686 ± 0.002	0.366 ± 0.005	1.00
		MHRM	/	/	/	/	0.00
		IWVAE	**0.691 ± 0.002**	0.469 ± 0.007	**0.747 ± 0.002**	**0.378 ± 0.005**	1.00
	Within	MCEM	0.571 ± 0.003	**0.530 ± 0.004**	0.598 ± 0.003	0.497 ± 0.004	1.00
		MHRM	/	/	/	/	0.00
		IWVAE	**0.608 ± 0.003**	0.495 ± 0.004	**0.683 ± 0.004**	**0.502 ± 0.004**	1.00
10000 500	Between	MCEM	0.670 ± 0.003	**0.498 ± 0.006**	0.713 ± 0.003	**0.376 ± 0.005**	1.00
		MHRM	/	/	/	/	0.00
		IWVAE	**0.691 ± 0.003**	0.428 ± 0.007	**0.756 ± 0.002**	0.357 ± 0.005	1.00
	Within	MCEM	0.580 ± 0.003	**0.542 ± 0.004**	0.606 ± 0.005	0.508 ± 0.004	1.00
		MHRM	/	/	/	/	0.00
		IWVAE	**0.625 ± 0.004**	0.488 ± 0.005	**0.717 ± 0.004**	**0.510 ± 0.005**	1.00

Table 8: Mean and standard error of **A** sparsity estimate on M3PL models under **double** regime setting. Factors are correlated. **Between** item structure: each item depends on 1 factor. **Within** item structure: each item depends on 2 factors
